# Downregulation of exosomal miR-204-5p and miR-632 as a biomarker for FTD: a GENFI study

**DOI:** 10.1136/jnnp-2017-317492

**Published:** 2018-02-06

**Authors:** Raphael Schneider, Paul McKeever, TaeHyung Kim, Caroline Graff, John Cornelis van Swieten, Anna Karydas, Adam Boxer, Howie Rosen, Bruce L Miller, Robert Laforce Jr, Daniela Galimberti, Mario Masellis, Barbara Borroni, Zhaolei Zhang, Lorne Zinman, Jonathan Daniel Rohrer, Maria Carmela Tartaglia, Janice Robertson

**Affiliations:** 1 Department of Laboratory Medicine and Pathobiology, University of Toronto, Toronto, Ontario, Canada; 2 Tanz Centre for Research in Neurodegenerative Diseases, University of Toronto, Toronto, Ontario, Canada; 3 Department of Computer Science, University of Toronto, Toronto, Ontario, Canada; 4 The Donnelly Centre for Cellular and Biomolecular Research, University of Toronto, Toronto, Ontario, Canada; 5 Department of Neurobiology, Karolinska Institute, Stockholm, Sweden; 6 Department of Neurology, Erasmus Medical Center, Rotterdam, The Netherlands; 7 Department of Neurology, University of California, San Francisco, California, USA; 8 Département des Sciences Neurologiques, Université Laval, Quebec, Canada; 9 Centro Dino Ferrari, Fondazione Ca’ Granda IRCCS Ospedale Policlinico, University of Milan, Milan, Italy; 10 LC Campbell Cognitive Neurology Research Unit, University of Toronto, Toronto, Ontario, Canada; 11 Sunnybrook Health Sciences Centre, University of Toronto, Toronto, Ontario, Canada; 12 Neurology Unit, Centre for Ageing Brain and Neurodegenerative Disorders, University of Brescia, Brescia, Italy; 13 Department of Molecular Genetics, University of Toronto, Toronto, Ontario, Canada; 14 Dementia Research Centre, University College London, London, UK; 15 Memory Clinic, University Health Network, Toronto, Ontario, Canada

## Abstract

**Objective:**

To determine whether exosomal microRNAs (miRNAs) in cerebrospinal fluid (CSF) of patients with frontotemporal dementia (FTD) can serve as diagnostic biomarkers, we assessed miRNA expression in the Genetic Frontotemporal Dementia Initiative (GENFI) cohort and in sporadic FTD.

**Methods:**

GENFI participants were either carriers of a pathogenic mutation in progranulin, chromosome 9 open reading frame 72 or microtubule-associated protein tau or were at risk of carrying a mutation because a first-degree relative was a known symptomatic mutation carrier. Exosomes were isolated from CSF of 23 presymptomatic and 15 symptomatic mutation carriers and 11 healthy non-mutation carriers. Expression of 752 miRNAs was measured using quantitative PCR (qPCR) arrays and validated by qPCR using individual primers. MiRNAs found differentially expressed in symptomatic compared with presymptomatic mutation carriers were further evaluated in a cohort of 17 patients with sporadic FTD, 13 patients with sporadic Alzheimer’s disease (AD) and 10 healthy controls (HCs) of similar age.

**Results:**

In the GENFI cohort, miR-204-5p and miR-632 were significantly decreased in symptomatic compared with presymptomatic mutation carriers. Decrease of miR-204-5p and miR-632 revealed receiver operator characteristics with an area of 0.89 (90% CI 0.79 to 0.98) and 0.81 (90% CI 0.68 to 0.93), respectively, and when combined an area of 0.93 (90% CI 0.87 to 0.99). In sporadic FTD, only miR-632 was significantly decreased compared with AD and HCs. Decrease of miR-632 revealed an area of 0.90 (90% CI 0.81 to 0.98).

**Conclusions:**

Exosomal miR-204-5p and miR-632 have potential as diagnostic biomarkers for genetic FTD and miR-632 also for sporadic FTD.

## Introduction

Frontotemporal dementia (FTD) is now recognised as the most common cause of early-onset dementia in people under the age of 60 years.^1^ FTD usually presents with either behavioural or language impairment. The pathogenic mechanisms resulting in FTD remain largely unknown, but current knowledge suggests that genetic, epigenetic and environmental factors contribute to disease development.^2^ Approximately 40% of patients with FTD have a positive family history of dementia^3^ and about 25% of patients with FTD have an identified genetic form of the disease.^1^ The vast majority of genetic FTD is inherited in an autosomal dominant pattern caused by mutations in one of three genes: chromosome 9 open reading frame 72 (*C9orf72*), progranulin (*GRN*) or microtubule-associated protein tau (*MAPT*). These genes provide an opportunity to study the disease in its presymptomatic phase and offer great hope for elucidating the pathogenic mechanisms that cause FTD. There is some mounting evidence that alterations in microRNA (miRNAs) may occur in FTD.^4–6^ MiRNAs are small, non-coding RNAs that regulate gene expression through post-transcriptional silencing of target mRNAs.^7^ The same miRNA may regulate hundreds of target mRNAs affecting complex disease pathways.^8^ MiRNAs are stable in body fluids and can be enriched in extracellular vesicles termed exosomes. These vesicles were thought to be a means for cells to discard unnecessary molecules into the extracellular space,^9^ but more recent studies have shown that cells can transfer proteins, lipids, DNA, RNA and miRNA to other cells via exosomes.^10^ Exosomes display different miRNA profiles compared with serum and cells, suggesting that a specific selection of exosomal miRNAs provides signals to regulate pathways in recipient cells.^11^ This intercellular transfer can influence a multitude of biological processes relevant to the nervous system such as neuronal survival, neurite outgrowth and synaptic plasticity.^12–15^ Disease-relevant miRNAs may be enriched within exosomes,^16^ and since miRNA expression can vary in different disease states, exosomal miRNAs are attractive targets for biomarker profiling.^17 18^

Genetic FTD is a rare condition, and single groups have only been able to study small numbers of patients. Through the Genetic Frontotemporal Dementia Initiative (GENFI), we obtained CSF from individuals who were either symptomatic or presymptomatic carriers of a known pathogenic mutation in *GRN*, *MAPT* or *C9orf72* or who were non-affected first-degree relatives of a known symptomatic carrier (healthy non-mutation carriers). We characterised miRNA expression profiles and found miR-204-5p and miR-632 significantly decreased in symptomatic compared with presymptomatic mutation carriers, suggesting low miR-204-5p and miR-632 as potential diagnostic biomarkers. In a separate cohort, we found miR-632 significantly decreased in sporadic FTD compared with sporadic Alzheimer’s disease (AD) and healthy controls (HCs), highlighting its potential as a diagnostic biomarker for sporadic FTD.

## Methods

### Ethics statements, sample collection and clinical data

Written informed consent and local research ethics boards’ approval was obtained. Six GENFI centres contributed CSF (Karolinska Institute, Department of Neurobiology, Stockholm, Sweden; Erasmus Medical Center, Department of Neurology, Rotterdam, The Netherlands; University College London, Dementia Research Centre, London, England; Université Laval, Département des Sciences Neurologiques, Quebec City, Canada; University of Milan, Centro Dino Ferrari, Fondazione Ca’ Granda IRCCS Ospedale Policlinico, Milan, Italy; and University of Toronto, Sunnybrook Health Sciences Centre, Toronto, Canada). The GENFI cohort consisted of 49 subjects: 38 mutation carriers (22 *GRN*, 11 *C9orf72* and 5 *MAPT*) and 11 first-degree relatives who tested negative for a mutation in the gene that had been found mutated in their affected first-degree relative (healthy non-mutation carriers). Twenty-three mutation carriers were presymptomatic, and 15 mutation carriers were symptomatic. The clinical presentation was behavioural variant FTD (bvFTD) (n=12), non-fluent variant primary progressive aphasia (nfvPPA) (n=1), semantic variant primary progressive aphasia (svPPA) (n=1) or dementia not otherwise specified (D-NOS) (n=1) (online supplementary table 1). Mini-Mental State Examination (MMSE^19^) was carried out in all individuals. A cohort of sporadic FTD, sporadic AD and HCs was recruited at the University Health Network Memory Clinic, Toronto, and the University of California San Francisco Memory and Aging Center. This sporadic disease cohort consisted of bvFTD (n=7), bvFTD/amyotrophic lateral sclerosis (ALS) (n=4), svPPA (n=3), nfvPPA/ALS (n=1), svPPA/ALS (n=1), nfvPPA (n=1), sporadic AD (n=13) and HCs (n=10) (online supplementary table 2). BvFTD met the Rascovsky diagnostic criteria,^20^ PPA met the Gorno-Tempini diagnostic criteria,^21^ ALS met the El Escorial diagnostic criteria^22^ and AD met the McKhann diagnostic criteria.^23^

10.1136/jnnp-2017-317492.supp1 Supplementary file 1

10.1136/jnnp-2017-317492.supp2 Supplementary file 2

### Samples for miRNA detection

Lumbar puncture was performed with a 20-gauge or 24-gauge spinal needle, and fluid was collected in polypropylene tubes according to local standards. Most sites follow ADNI procedures manual (http://www.adni-info.org/). CSF was stored in aliquots at −80°C until use.

### Real-time PCR

For the genetic cohort (n=49), 500 µL of each CSF sample was thawed and centrifuged at 10 000 × g for 5 min to pellet any debris. To isolate exosomes, the supernatant was transferred to a new reaction vial, and 200 µL precipitation buffer (miRCURY Exosome Isolation Kit, Exiqon, Copenhagen, Denmark) was mixed with the supernatant. The mix was incubated at 4°C for 60 min and spun for 30 min at 10 000 × g at 20°C. The supernatant was discarded, and lysis buffer containing synthetic spike-ins (UniSp2, UniSp4 and UniSp5) was added to the pellet. RNA was extracted using spin column chromatography (miRCURY RNA Isolation Kit, Exiqon). To obtain cDNA, each RNA sample was incubated for 60 min at 42°C in the presence of reaction buffer, nuclease-free water, enzyme mix and synthesis RNA spike-in mix (cel-miR-39-3p and UniSp6) (miRCURY RNA Isolation Kit, Exiqon). Reverse transcriptase (RT) was heat-inactivated for 5 min at 95°C, and the cDNA samples were immediately stored at −80°C. Immediately prior to real-time PCR, each cDNA sample was thawed and added to a Master Mix working-solution containing SYBR Green (Exiqon). Ten microlitres of this mix was added to each of the 768 wells of the ready-to-use Human microRNA panel I+II, V4.M (Exiqon). Panel I+II contained a total of 752 individual miRNA primer sets plus control assays. Plates were spun at 1500 × g for 1 min. Plates were run on the Applied Biosystems 7900HT Real-Time PCR System (Thermo Fisher Scientific, Waltham, Massachusetts, USA). Only miRNAs detected with Ct <40 were included in the analysis. After normalisation to cel-mir-39-3p, as previously described by Freischmidt *et al*,^24^ cycle threshold (Ct) values were converted to linear scale relative to the control group (healthy non-mutation carriers), and Log2 conversion was applied (Exiqon Data Analysis Guide for miRCURY GenEx software v 3). For the sporadic disease cohort (n=40), CSF was thawed, and cDNA was obtained from the exosomal miRNA content as described above. For technical validation of the results obtained in the GENFI cohort and for the sporadic disease cohort, a Master Mix working-solution containing either hsa-204-5p or hsa-miR-632 PCR primer set (both Exiqon) and SYBR Green (Exiqon) was prepared. Master Mix and samples were added to 96-well plates and run on the Applied Biosystems Step One Plus Real-Time PCR System (Thermo Fisher Scientific). MiRNA expression changes were calculated relative to HCs using the 2^−ΔΔCt^ method^25^ with ^Δ^Ct=Ct_miRNA_ – Ct_reference_ and ^ΔΔ^Ct=^Δ^Ct_patient or mutation carrier_ − ^Δ^Ct_control pool_. UniSp6 spike-in was used as a reference for normalisation. RNA and DNA spike-ins showed steady levels across samples indicating accurate RT reaction and PCR. Applied Biosystems SDS V.2.2.2. software (Thermo Fisher Scientific) and GenEx 6 (MultiD Analyses, Göteborg, Sweden) were used for miRNA expression processing prior to statistical analysis.

### Statistical analysis

Welch’s t-test was performed and corrected for multiple comparisons using the Holm-Sidak method when relative miRNAs expression changes passed D’Agostino & Pearson normality test. When relative miRNA expression changes calculated as 2^−ΔΔCt^ were not normally distributed, Mann-Whitney U test was performed. Fisher’s exact test was used to detect differences in miRNA detection frequency. Correlations between clinical data and miRNA expression were calculated using Spearmans’s rank order correlation. Receiver operating characteristics (ROC) curves and the area under the curve (AUC) were established to evaluate the diagnostic value of miRNA expression changes. For cross-validation, we used 50% of the dataset to train linear models and 50% to validate the results. We then calculated Pearson’s bivariate correlation. Statistical analysis was performed using GraphPad Prism V.7.01 (La Jolla, California, USA). IBM SPSS V.24.0 was used for logistic regression, ROC calculations and cross-validation. P values <0.05 were considered significant. When the 90% CI included 1, P values were reported as P trend.

### Target prediction and gene ontology analysis

Targets of each significantly different miRNA were predicted using miRWalk 2.0, which combines information from 12 existing miRNA-target prediction programs (DIANA-microTv4.0, DIANA-microT-CDS, miRanda-rel2010, mirBridge, miRDB4.0, miRmap, miRNAMap, doRiNA/PicTar2, PITA, RNA22v2, RNAhybrid2.1 and Targetscan6.2)^26^ (online supplementary table 3). Only experimentally validated mRNAs were included in further analyses. The KEGG database was used to identify target mRNAs in biological pathways (c2.cp.kegg.v5.1.symbols.gmt).^27^ To assess whether target mRNAs were previously found highly expressed in the human frontal and temporal lobes relative to the entire human brain, we searched the Allen Brain Atlas (http://www.brain-map.org).^28^ The original search can be reproduced at http://human.brain-map.org/microarray/search/show?domain1=4005&domain2=4009,4132&selected_donors=9861,10021,12876,14380,15496,15697&search_type=differential. FunRich V.3.0 was used to generate Venn diagrams of validated targets found with miRWalk 2.0, the KEGG pathway database and the Allen Brain Atlas.

10.1136/jnnp-2017-317492.supp3 Supplementary file 3

## Results

### Exosomal miR-204-5p and miR-632 expression is low in genetic FTD

**Figure 1:**
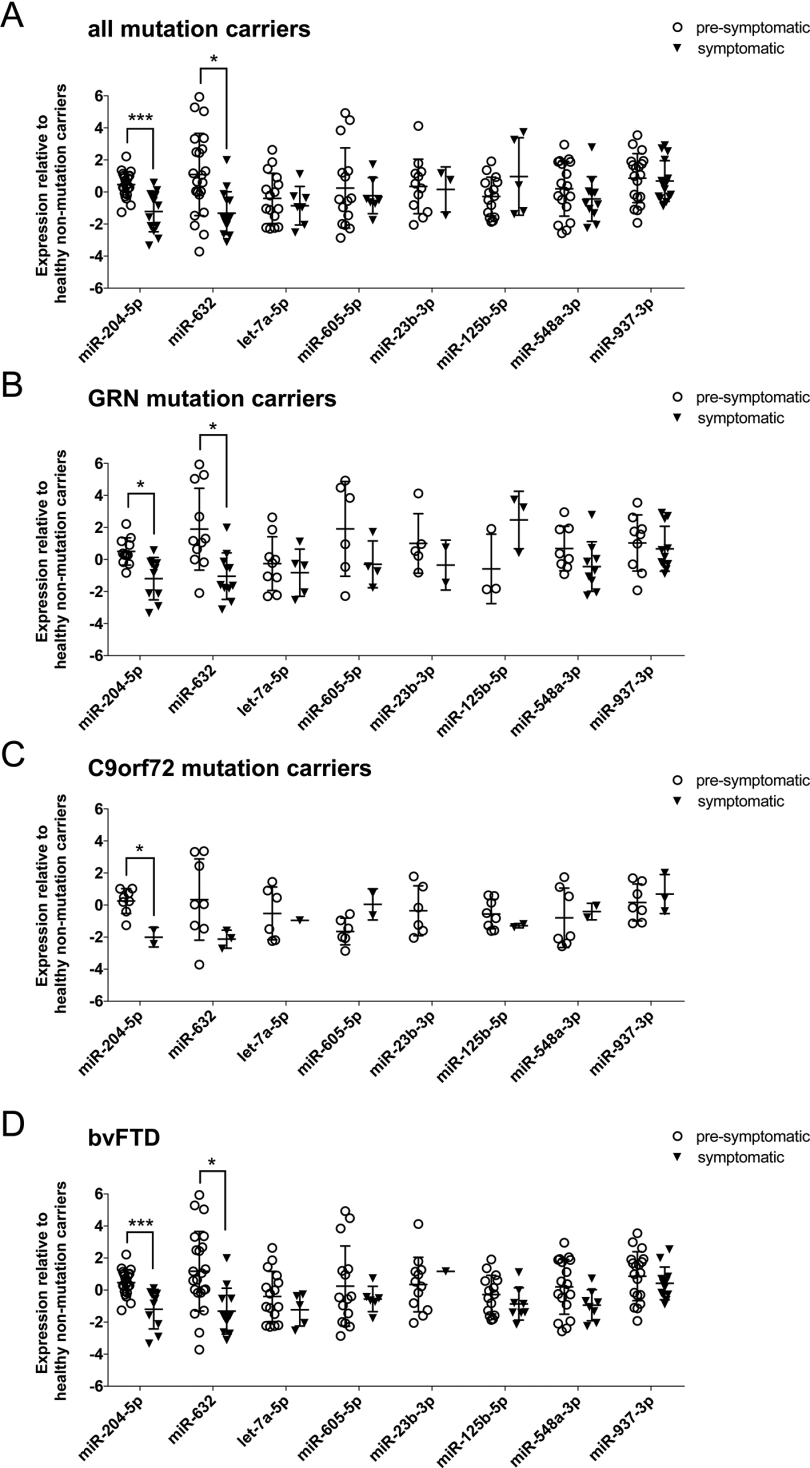
Relative expression of miR-204-5p and miR-632 is lower in symptomatic compared with presymptomatic mutation carriers. MiRNA expression was calculated relative to that of healthy non-mutation carriers. Data from individuals with any of the three mutations were grouped, and expression of both miR-204-5p and miR-632 was found to be significantly lower in symptomatic compared with presymptomatic individuals (A). Only data from individuals with a *GRN* mutation were grouped, and expression of both miR-204-5p and miR-632 was found to be significantly lower in symptomatic compared with presymptomatic individuals (B). Only data from individuals with a *C9orf72* mutation were grouped, and expression of miR-204-5p was found to be significantly lower in symptomatic compared with presymptomatic individuals (C). Only data from individuals with any of the three mutations and the bvFTD phenotype were grouped, and expression of both miR-204-5p and miR-632 was found to be significantly lower in symptomatic compared with presymptomatic individuals (D). Welch’s t-tests were corrected for multiple comparisons using the Holm-Sidak method, *P<0.05, ***P<0.005. Mean and SD of mean are shown. *C9orf72*, chromosome 9 open reading frame 72; *GRN*, progranulin; bvFTD, behavioural variant FTD.

We reasoned that a clinically useful diagnostic biomarker would be detectable in healthy individuals and altered in disease. We found two miRNAs (miR-204-5p and miR-632) in all exosomal CSF samples of healthy non-mutation carriers and an additional six miRNAs in at least 70% (miR-605-5p, let-7a-5p, miR-548a-3p, miR-23b-3p, miR-125b-5p and miR-937-3p) (online supplementary figure 1). MiRNA expression in exosomal CSF samples from healthy non-mutation carriers was used to obtain baseline values for each miRNA. MiRNA expression relative to this baseline was compared between presymptomatic and symptomatic mutation carriers. No significant expression changes were found between healthy non-mutation carriers and presymptomatic mutation carriers. Relative expression of both miR-204-5p and miR-632 was significantly lower in symptomatic compared with presymptomatic mutation carriers (P<0.005 and P<0.05) (figure 1A). Relative expression of miR-204-5p was significantly lower in symptomatic mutation carriers with either *GRN* or *C9orf72* mutations (P<0.05 and P<0.05) (figure 1B,C). Relative expression of miR-632 was significantly lower in symptomatic compared with presymptomatic mutation carriers in the *GRN* group (P<0.05) but not in the *C9orf72* group (figure 1B,C). With only one symptomatic mutation carrier in the *MAPT* group, statistical analysis was not possible. Most symptomatic mutation carriers had been diagnosed with bvFTD (80%) (online supplementary table 1). Relative expression of both miR-204-5p and miR-632 was still significantly lower when bvFTD only was compared with presymptomatic mutation carriers (P<0.005 and P<0.05) (figure 1D). Technical validation using individual primer sets showed decrease of miR-204-5p and miR-632 similar to the results obtained with the miRNA panels, when the relative transcript number was compared with the pooled sample of healthy non-mutation carriers (online supplementary figure 2A–D). Raw Ct values of both miRNAs were significantly higher in symptomatic mutation carriers, indicating decreased expression in symptomatic individuals, independent of normalisation (online supplementary figure 2E). Only one individual was diagnosed with either svPPA, nfvPPA or D-NOS; therefore, statistical analysis of these clinical phenotypes was not possible. Age was significantly different between groups with symptomatic mutations carriers being older than presymptomatic mutation carriers (all mutation carriers: P<0.0001, *GRN* mutation carriers: P<0.005, *C9orf72* mutation carriers: P<0.05 and bvFTD: P<0.0001). Notably, there was no correlation between miR-204-5p expression and age in healthy non-mutation carriers and HCs, and there was a modest increase of miR-632 expression with age in these healthy individuals (P<0.05) (online supplementary figure 3). When we analysed females and males separately, we found a decrease of 204-5p and miR-632 in symptomatic compared with presymptomatic female mutation carriers (n=25), before correcting for multiple comparisons (P<0.005 and P<0.05). The numbers of male mutation carriers was smaller (n=13), and comparing miR-204-5p and miR-632 between symptomatic and presymptomatic male mutation carriers only revealed a trend towards significances, before correction for multiple comparisons (P<0.06 and P<0.07) (data not shown). We did not observe a significant change of miR-204-5p or miR-632 relative to disease duration or MMSE results in healthy non-mutation carriers, presymptomatic or symptomatic individuals (data not shown).

10.1136/jnnp-2017-317492.supp4 Supplementary file 4

10.1136/jnnp-2017-317492.supp5 Supplementary file 5

10.1136/jnnp-2017-317492.supp6 Supplementary file 6

### Low exosomal miR-204-5p and miR-632 expression distinguishes symptomatic from presymptomatic individuals

**Figure 2:**
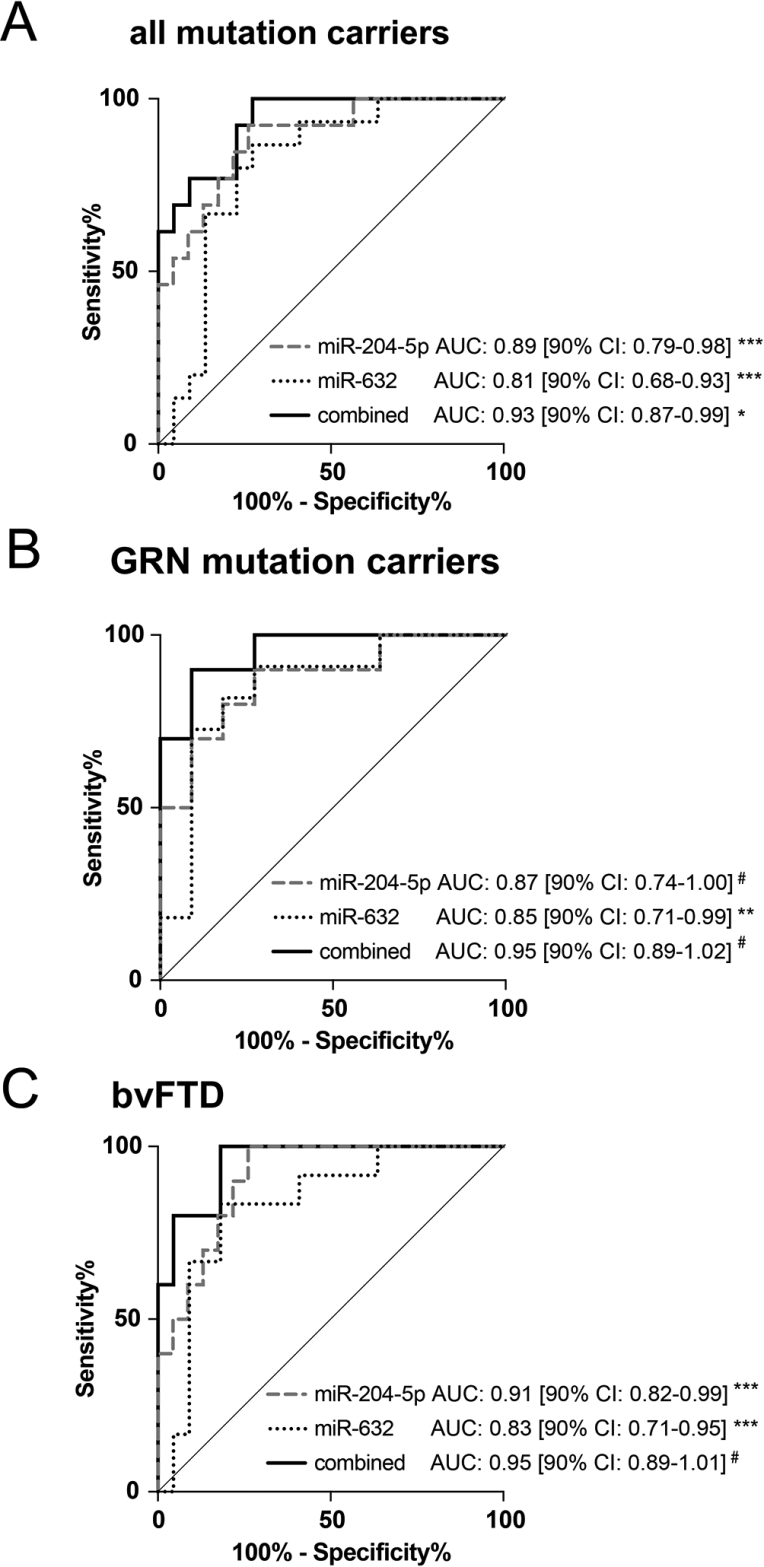
ROC curve analysis discriminates symptomatic from presymptomatic mutation carriers. MiR-204-5p and miR-632 expression can discriminate between presymptomatic and symptomatic individuals based on ROC. 90% CI are reported in brackets. Dashed grey lines represent miR-204-5p, dotted black lines represent miR-632 and solid black lines represent the combination of miR-204-5p and miR-632 determined by logistic regression. All mutation carriers (A), *GRN* mutation carriers (B) and bvFTD phenotype (C) were analysed separately, *P<0.05, **P<0.01, ***P<0.005. ^#^P trend CI includes 1.0. AUC, area under the curve; *GRN*, progranulin; bvFTD, behavioural variant FTD.

To assess whether the changes in miR-204-5p and miR-632 expression can distinguish symptomatic from presymptomatic individuals, we calculated ROC. We found that a decrease of miR-204-5p and miR-632 discriminated well between presymptomatic and symptomatic individuals. The AUC for miR-204-5p was 0.89 (90% CI 0.79 to 0.98) (P<0.005), and the AUC for miR-632 was 0.81 (90% CI 0.68 to 0.93) (P<0.005). Combination of miR-204-5p and miR-632 narrowed the CI and increased the AUC to 0.93 (90% CI 0.87 to 0.99) (P<0.05) (figure 2A). In the *GRN* group, miR-632 discriminated well between presymptomatic and symptomatic individuals with an AUC of 0.85 (90% CI 0.71 to 0.99) (P<0.01), and there was a trend for miR-204-5p and the combination of miR-204-5p and miR-632 (figure 2B). In the *C9orf72* group, only three individuals were symptomatic, and ROC analysis did not yield significant results (data not shown). For patients with bvFTD, miR-204-5p and miR-632 discriminated well between presymptomatic and symptomatic individuals with AUCs of 0.91 (90% CI 0.82 to 0.99) and 0.83 (90% CI 0.71 to 0.95) (both P<0.005), and there was a trend for the combination of miR-204-5p and miR-632 (figure 2C). In a cross-validation analysis, low miR-204-5p correlated significantly with symptomatic status (Pearson correlation r=0.636 and P<0.05 in both the training and validation dataset), while low miR-632 did not significantly correlate with symptomatic status in our model.

### Specific exosomal miRNAs are detected less or more commonly in FTD

We hypothesised that certain miRNAs would not be decreased or increased but undetectable in either health or disease. We compared symptomatic mutation carriers (symptomatic) with healthy non-mutation carriers and presymptomatic mutation carriers (healthy) to assess for differences between disease and health, regardless of mutation status. Comparing the frequency of detected miRNAs between symptomatic and healthy participants, we found miR-23b-3p, miR-326, miR-877-5p, miR-892a less commonly (P<0.05) and miR-708-3p more commonly (P<0.01) in symptomatic compared with healthy participants (table 1). When we compared presymptomatic with symptomatic mutation carriers, we found miR-30b-5p and miR-373-3p less commonly in the *GRN* group (P<0.05) (table 1). No significant differences were found between presymptomatic and symptomatic carriers of *C9orf72* or *MAPT* mutations.

**Table 1:** Certain miRNAs were less frequently detected in a subgroup of study participants

miRNA	Group	Healthy		Symptomatic		P value	Comment
		Detectable	Undetectable	Detectable	Undetectable		
miR-23b-3p	All participants (n=49)	20	14	3	12	P<0.05	Less common in symptomatic
miR-326	All participants (n=49)	8	26	0	15	P<0.05	Less common in symptomatic
miR-877-5p	All participants (n=49)	14	20	1	14	P<0.05	Less common in symptomatic
miR-892a	All participants (n=49)	8	26	0	15	P<0.05	Less common in symptomatic
miR-708-3p	All participants (n=49)	21	13	15	0	P<0.01	More common in symptomatic
miR-30b-5p	*GRN* mutation carriers (n=22)	7	4	1	10	P<0.05	Less common in symptomatic
miR-373-3p	*GRN* mutation carriers (n=22)	8	3	2	9	P<0.05	Less common in symptomatic

*P<0.05, **P<0.01.

The detection frequency of miRNAs was compared between healthy non-mutation carriers and presymptomatic mutation carriers (healthy) and symptomatic mutation carriers (symptomatic) using Fisher’s exact test. MiR-23b-3p, miR-326, miR-877-5p and miR-892a were detected less commonly and miR-708-3p more commonly in symptomatic compared with presymptomatic mutation carriers and healthy non-mutation carriers. The detection frequency of miRNAs was compared between presymptomatic mutation carriers and symptomatic mutation carriers using Fisher’s exact test. MiR-30b-5p and miR-373-3p were detected less commonly in symptomatic compared with presymptomatic *GRN* mutation carriers.

*GRN*, progranulin.

### Expression of exosomal miR-632 is lower in sporadic FTD compared with sporadic AD and HCs

**Figure 3:**
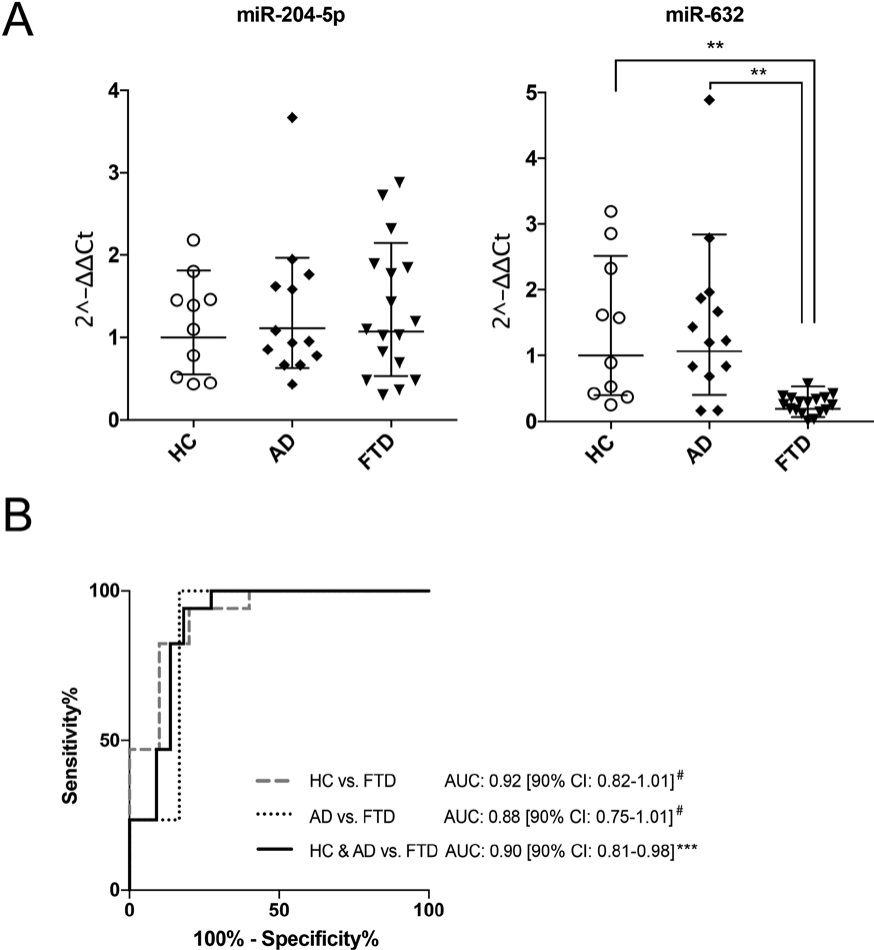
Relative expression of miR-632 is lower in sporadic FTD compared with sporadic AD and HCs. MiRNA expression was calculated relative to that of HCs using the 2^−ΔΔCt^ method. Expression values of HCs, sporadic AD and sporadic FTD were compared. Expression of miR-204-5p was similar between groups (A, left), and expression of miR-632 was significantly lower in FTD using Welch’s t-tests (A, right). MiR-632 expression can discriminate between FTD and non-FTD (HC and AD) based on ROC. 90% CI reported in brackets. Dashed grey line represents HC versus FTD, dotted black line represents AD versus FTD and solid black line represents the combination of HC and AD determined by logistic regression (B). **P<0.01, ***P<0.005, ^#^P trend CI includes 1.0. Mean and SD of mean are shown. AUC, area under the curve; AD, Alzheimer’s disease; FTD, frontotemporal dementia; HC, healthy control.

We next sought to validate miR-204-5p and miR-632 as biomarker candidates in a cohort of sporadic FTD. We observed no significant decrease of miR-204-5p expression in sporadic FTD compared with sporadic AD or HCs of similar age (figure 3A, left); however, mir-632 was significantly decreased in sporadic FTD compared with HCs or patients with AD (P<0.005) (figure 3A, right). There was no significant difference between FTD phenotypes (bvFTD, bvFTD/ALS, svPPA, nfvPPA/ALS, svPPA/ALS, nfvPPA) (data not shown). To evaluate the diagnostic value of miR-632 in differentiating sporadic FTD from AD and HCs, we constructed ROC curves (figure 3B). When FTD was compared with all non-FTD (HC and AD), the AUC was 0.90 (90% CI 0.81 to 0.98) (P<0.005). There was a trend for AUC to distinguish FTD from HC or AD separately (figure 3B). In a cross-validation analysis, low miR-632 correlated significantly with a diagnosis of FTD (Pearson correlation r=0.578 and P<0.05 in both the training and validation dataset).

### Decrease or loss of miRNA may result in disease-relevant pathway activation

**Figure 4:**
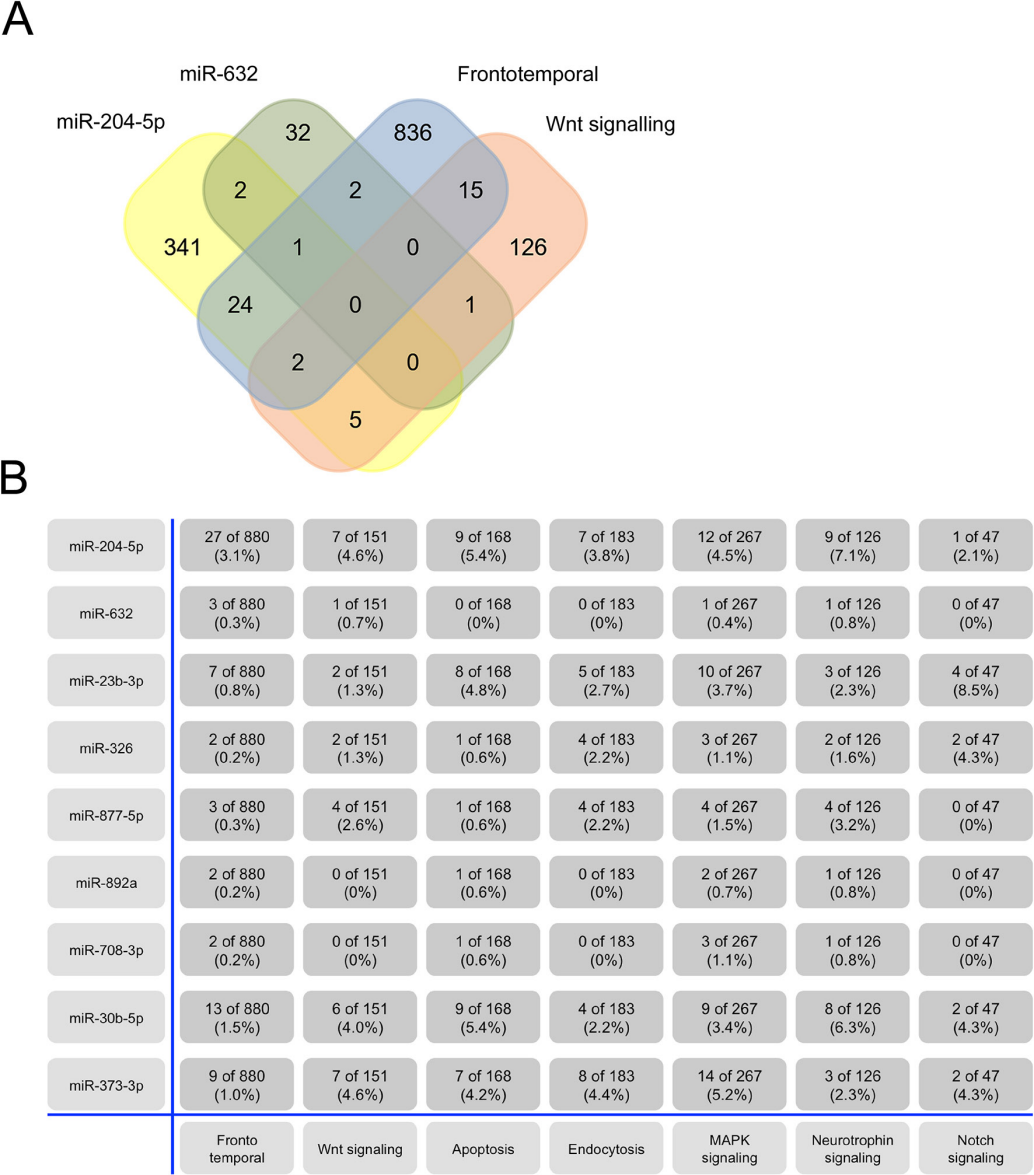
Venn diagrams of miRNA targets. Overlap of mRNA targeted by miRNAs found decreased in symptomatic compared with presymptomatic mutation carriers (miR-204-5p and miR-632), mRNAs enriched in the human frontal and temporal lobes (frontotemporal) and mRNAs implicated in Wnt signalling (A). Overlap of mRNA targeted by miRNAs less frequently detectable in FTD (miR-23b-3p, miR-326, miR-877-5p, miR-892a, miR-30b-5p and miR-373-3p), more frequently detected in FTD (miR-708-3p), mRNAs enriched in the human frontal and temporal lobes and mRNAs implicated in Wnt signalling, apoptosis, MAPK signalling, endocytosis, notch signalling or neurotrophin signalling (B). Number of validated targets within a pathway are shown in grey cells, and % of targets found in each pathway are shown in brackets below.

Since the main function of miRNAs is silencing of mRNA, we identified mRNA targeted by miRNAs found downregulated (miR-204-5p and miR-632), less commonly (miR-23b-3p, miR-326, miR-877-5p, miR-892a, miR-30b-5p and miR-373-3p) or more commonly in FTD (miR-708-3p) (online supplementary table 3). We found 375 mRNAs targeted by miR-204-5p and 38 mRNAs targeted by miR-632, including three mRNAs targeted by both miRNAs (HRK, KNTC1 and POU2F1) (figure 4A). When we compared, this group of target mRNAs with mRNAs enriched in the human frontal and temporal lobes (Allen Institute, http://www.brain-map.org),^28^ we found HRK, a central mediator of apoptosis,^29^ to be a potential target of both miR-204-5p and miR-632 (figure 4A). Wnt signalling has been implicated as a central disease pathway in FTD with *GRN* mutations.^30 31^ One of the mRNAs targeted by miR-204-5p (FZD8) in the Wnt signalling pathway was highly expressed in the human frontal and temporal lobes. Each miRNA we found downregulated (miR-204-5p and miR-632) or less frequently in FTD (miR-23b-3p, miR-326, miR-877-5p, miR-892a, miR-30b-5p and miR-373-3p) targets several mRNA enriched in the human frontal and temporal lobes (figure 4B). Interestingly, targets of miR-204-5p and of the two miRNAs less frequent detected in symptomatic *GRN* mutation carriers (miR-30b-5p and miR-373-3p) were relatively enriched in the frontal and temporal lobes (27, 13 and 9 targets). In addition to Wnt signalling, RNA targets of exosomal miRNAs were found in apoptosis, MAPK signalling, endocytosis, notch signalling and neurotrophin signalling (figure 4B).

## Discussion

Discovery of biomarkers for FTD would result in more accurate diagnoses and facilitate early and specific treatment efforts. Previous studies indicate altered expression of specific miRNAs in the brains of patients affected by neurodegenerative diseases including FTD, AD, Parkinson’s disease and Huntington’s disease.^5 32 33^ Galimberti *et al*^34^ measured miRNAs in both serum and CSF and found both miR-125b and miR-26b significantly decreased in AD. More recently, Sørensen *et al*^35^ found let-7i-5p and miR-15a-5p increased and miR-29c-3p decreased in CSF samples from patients with AD. Since disease-relevant miRNAs may be enriched within exosomes,^17 18^ we opted to evaluate exosomal miRNA. We found significantly lower expression of miR-204-5p and miR-632 in symptomatic compared with presymptomatic mutation carriers in the genetic FTD cohort. While the *C9orf72* group followed this trend, most data supporting our conclusions come from symptomatic *GRN* mutation carriers and individuals diagnosed with bvFTD.

In the sporadic disease cohort, miR-204-5p expression was not significantly different in FTD compared with AD and HCs, suggesting that genetic factors influence miR-204-5p expression. However, miR-632 was significantly decreased in sporadic FTD, underlining its potential as a diagnostic biomarker candidate for both genetic and sporadic FTD. ROC discriminated well between FTD and non-FTD (HC and AD). We appreciate that the frequency distribution of FTD, AD and HCs in our sample was not necessarily representative and that the true sensitivity and specificity of the test may be lower in a typical clinical setting.

Using in silico analysis, we found HRK to be a potential target of both miR-204-5p and miR-632 in the human frontal and temporal lobes. HRK encodes for the apoptosis activator, HARAKIRI.^29^ Since the main function of miRNAs is silencing of mRNA, low miR-204-5p and miR-632 could result in pathologically increased HRK and apoptosis leading to degenerative changes within the frontal and temporal lobes of FTD patients. Wnt signalling has been implicated in FTD with *GRN* mutations,^30 31^ and targeting the Wnt signalling pathway may emerge as a future therapeutic.^36^ In addition to apoptosis and Wnt signalling, mRNA targets were found in other biological pathways that have been linked with neurodegeneration and/or FTD such as MAPK signalling,^37^ endocytosis,^38 39^ notch signalling^40^ and neurotrophin signalling.^41 42^

In summary, we showed exosomal miR-204-5p and miR-632 to have potential as diagnostic biomarkers for genetic FTD and miR-632 also for sporadic FTD. Through in silico target prediction and disease pathway analysis, we found some of these miRNAs to target mRNAs involved in pathways previously linked to FTD. To our knowledge, none of the miRNAs we found significantly altered in CSF exosomes have previously been reported in FTD or been implicated in its pathology.^5^ Since miRNAs are still in their infancy, this is not unexpected. We must consider some limitations of the current study. We appreciate that sex was not matched in all groups. For example, while presymptomatic and symptomatic *GRN* groups contained equal numbers of females and males in the respective groups, all symptomatic *C9orf72* mutation carriers were male, which may have introduced bias. Furthermore, most presymptomatic and symptomatic mutation carriers tested positive for a mutation in *GRN*, so our results will have to be confirmed in larger cohorts including more patients with *C9orf72* and *MAPT* mutations. Ideally, our results will be confirmed in prospective studies including cohorts of genetic and sporadic FTD before the miRNA expression changes described here would be used in clinical practice. For the time being, our findings highlight that exosomal miRNAs have potential as diagnostic biomarkers for genetic and sporadic FTD.
