# Supplementary material for: Downregulation of exosomal miR-204-5p and miR-632 as a biomarker for FTD: a GENFI study
Source: J Neurol Neurosurg Psychiatry. 2018 Feb 6;89(8):851–8. doi: 10.1136/jnnp-2017-317492 (PMC6045452; doi:10.1136/jnnp-2017-317492)
Supplement: Supplementary file 1 [file jnnp-2017-317492supp001.pdf]

Supplementary table 1

case#	age (years)	sex	clinical phenotype	type of mutation	test result
1	62	F	bvFTD	*GRN (p.Val200GlyfsX18)*	positive
2	56	F	bvFTD	*GRN (p.Tyr294X)*	positive
3	62	F	bvFTD	*GRN (p.Tyr294X)*	positive
4	72	M	D-NOS	*GRN (p.Tyr294X)*	positive
5	57	F	bvFTD	*GRN (p.Asn118PhefsX4)*	positive
6	60	M	bvFTD	*GRN (G35fs)*	positive
7	63	F	bvFTD	*GRN (G35fs)*	positive
8	57	F	bvFTD	*GRN (G35fs)*	positive
9	66	F	nfvPPA	*GRN (G35fs)*	positive
10	78	F	bvFTD	*GRN (C157fs)*	positive
11	72	M	bvFTD	*GRN (C149fs)*	positive
12	59	F	pre-symptomatic	*GRN (V411fs)*	positive
13	50	F	pre-symptomatic	*GRN (S82fs)*	positive
14	57	M	pre-symptomatic	*GRN (Q125X)*	positive
15	56	F	pre-symptomatic	*GRN(S82fs)*	positive
16	45	M	pre-symptomatic	*GRN (G35fs)*	positive
17	53	F	pre-symptomatic	*GRN (G35fs)*	positive
18	34	F	pre-symptomatic	*GRN (G35fs)*	positive
19	55	F	pre-symptomatic	*GRN (G35fs)*	positive
20	50	M	pre-symptomatic	*GRN (G35fs)*	positive
21	42	F	pre-symptomatic	*GRN (G35fs)*	positive
22	57	F	pre-symptomatic	*GRN (C416fs)*	positive
23	66	F	healthy non-mutation carrier	*GRN (C416fs)*	negative
24	37	M	healthy non-mutation carrier	*GRN (G35fs)*	negative
25	58	F	healthy non-mutation carrier	*GRN (G35fs)*	negative
26	52	M	healthy non-mutation carrier	*GRN (W386X)*	negative
27	55	F	healthy non-mutation carrier	*GRN (W386X)*	negative
28	75	M	svPPA	*C9orf72*	positive
29	79	M	bvFTD	*C9orf72*	positive
30	54	M	bvFTD	*C9orf72*	positive
31	42	F	pre-symptomatic	*C9orf72*	positive
32	46	F	pre-symptomatic	*C9orf72*	positive
33	65	F	pre-symptomatic	*C9orf72*	positive
34	45	M	pre-symptomatic	*C9orf72*	positive
35	46	F	pre-symptomatic	*C9orf72*	positive
36	51	F	pre-symptomatic	*C9orf72*	positive
37	48	M	pre-symptomatic	*C9orf72*	positive
38	42	M	pre-symptomatic	*C9orf72*	positive
39	26	M	healthy non-mutation carrier	*C9orf72*	negative
40	46	F	healthy non-mutation carrier	*C9orf72*	negative
41	56	F	bvFTD	*MAPT (IVS10+16)*	positive
42	33	F	pre-symptomatic	*MAPT (P301L)*	positive
43	47	M	pre-symptomatic	*MAPT (P301L)*	positive
44	45	F	pre-symptomatic	*MAPT (P301L)*	positive
45	28	F	pre-symptomatic	*MAPT (IVS10+16)*	positive
46	42	M	healthy non-mutation carrier	*MAPT (P301L)*	positive
47	43	F	healthy non-mutation carrier	*MAPT (P301L)*	negative
48	64	F	healthy non-mutation carrier	*MAPT (IVS10+16)*	negative
49	26	M	healthy non-mutation carrier	*MAPT (IVS10+16)*	negative
