# Supplementary material for: Downregulation of exosomal miR-204-5p and miR-632 as a biomarker for FTD: a GENFI study
Source: J Neurol Neurosurg Psychiatry. 2018 Feb 6;89(8):851–8. doi: 10.1136/jnnp-2017-317492 (PMC6045452; doi:10.1136/jnnp-2017-317492)
Supplement: Supplementary file 2 [file jnnp-2017-317492supp002.pdf]

## Supplementary table 2

| case# | age (years) | sex | clinical phenotype |
|---|---|---|---|
| 50 | 72 | M | HC |
| 51 | 79 | M | HC |
| 52 | 72 | M | HC |
| 53 | 71 | F | HC |
| 54 | 73 | M | HC |
| 55 | 67 | M | HC |
| 56 | 60 | F | HC |
| 57 | 70 | F | HC |
| 58 | 56 | F | HC |
| 59 | 65 | F | HC |
| 60 | 72 | F | AD |
| 61 | 77 | M | AD |
| 62 | 59 | F | AD |
| 63 | 55 | M | AD |
| 64 | 67 | M | AD |
| 65 | 60 | M | AD |
| 66 | 60 | F | AD |
| 67 | 62 | M | AD |
| 68 | 63 | F | AD |
| 69 | 61 | M | AD |
| 70 | 58 | M | AD |
| 71 | 67 | F | AD |
| 72 | 63 | M | AD |
| 73 | 74 | M | bvFTD/ALS |
| 74 | 63 | M | svPPA |
| 75 | 74 | M | bvFTD |
| 76 | 60 | F | nfvPPA/ALS |
| 77 | 78 | M | bvFTD |
| 78 | 50 | M | bvFTD/ALS |
| 79 | 69 | M | bvFTD |
| 80 | 64 | M | bvFTD |
| 81 | 70 | M | svPPA |
| 82 | 60 | M | bvFTD/ALS |
| 83 | 56 | M | nfvPPA |
| 84 | 69 | F | bvFTD |
| 85 | 80 | M | svPPA/ALS |
| 86 | 76 | F | bvFTD |
| 87 | 65 | M | bvFTD/ALS |
| 88 | 69 | F | svPPA |
| 89 | 57 | M | bvFTD |
