# Supplementary material for: Downregulation of exosomal miR-204-5p and miR-632 as a biomarker for FTD: a GENFI study
Source: J Neurol Neurosurg Psychiatry. 2018 Feb 6;89(8):851–8. doi: 10.1136/jnnp-2017-317492 (PMC6045452; doi:10.1136/jnnp-2017-317492)
Supplement: Supplementary file 3 [file jnnp-2017-317492supp003.pdf]

Supplementary table 3

Frontotemporal	Wnt signalling	Apoptosis	Endocytosis	MAPK signalling	Neurotrophin signalling	Notch signalling		miR-204-5p	miR-632	miR-23b-3p	miR-326	miR-877-5p	miR-892a	miR-708-3p	miR-30b-5p	miR-373-3p
TMEM155	JUN	CASP10	F2R	JUN	JUN	HES5		ZWILCH	PRDX6	SSRP1	ZNF394	USP2	ELP2	CRLF3	RUNX2	FAM160B1
FOXG1	LRP5	CASP9	EPN3	MEF2C	FRS2	DTX3		BRD2	HRK	CEPT1	WBSCR27	CDKN1B	BNC2	FGF2	SIKE1	ZNF514
LY86-AS1	LRP6	CASP8	IQSEC1	ELK4	CALM2	NOTCH4		RAB10	NR6A1	POM121C	ABCC6	VDAC1	OBFC1	RAP2B	CHD1	LMNB1
A_24_P895687	PPP3R2	CASP7	IQSEC3	ELK1	CALM1	DTX3L		KIAA1462	IFNGR2	PPIC	ABCC6	UBE2N	NARS	RAD51AP1	LIN28B	GPR155
CCK	SFRP2	CHUK	ADRBK2	JUND	PDK1	NOTCH3		STEAP4	SOX5	EMC1	MTHFD2	PTGFRN	LYRM7	42986	ZNF460	PIGA
CCK	SFRP1	PRKAR2B	ADRBK1	GADD45B	RPS6KA6	NOTCH2		SLC16A5	PPP2R1B	CDCA8	PKM	EIF1AX	LYRM7	CCDC169	RNF135	EIF2B2
GDA	PPP3CC	TNF	ADRB3	ZAK	TP73	EP300		SOD2	PLEKHG4	SESN2	BCL11B	GRB10	ALG9	DENND5B	MATR3	SH3GLB1
THEMIS	VANGL1	TNFSF10	ADRB2	STMN1	MAP3K5	HES1		CDH2	ATP5C1	ZNF319	ZNF689	INPP4A	CYLC2	42986	ZXDB	GTF2IRD2B
HSPB3	PPP3R1	BIRC3	EPN2	RRAS2	RPS6KA5	NOTCH1		CAMK2G	HRK	STAMBPL1	ACTG1	COLEC12	CBY3	KLHL15	CLDN12	CCND2
NRGN	FZD1	XIAP	STAMBP	MAP3K5	BRAF	NUMB		ANKRD45	PET117	TSNAX	ARL5B	MAPK8	PLAGL2	HNRNPA2B1	FANCL	MASTL
A_32_P21848	FZD4	PPP3R2	ADRB1	MAP3K1	MAP3K1	PSEN2		GALNT10	THYN1	PRDX1	EPHB3	RNF167	PGD	PHACTR2	SKIDA1	RELA
SLC30A3	APC2	PPP3CC	IGF1R	MAP3K3	MAP3K3	PSEN1		ZDHHC20	FOXF2	TAB3	SLC47A1	AMOTL2	KATNAL1	G2E3	MTDH	MED18
TESPA1	FZD6	PPP3R1	CLTCL1	MAP3K4	IKBKB	PTCRA		MYOC	PTGS1	ZNF281	SLC47A1	IGF2	RHOG	FGF2	PPP1R12B	NR2F2
PKD2L1	FZD7	MYD88	HGS	MAP3K7	ABL1	SNW1		MEIS1	ZNF417	HMGB2	A4GALT	SLC25A5	KIF5B	GP5	GOLGA8B	NUDT3
A_32_P197156	SENP2	FADD	VPS37C	MAP3K8	AKT1	APH1A		AKAP13	MAPKAPK2	EEF1A1	DYNLT1	SMCHD1	IL6ST	TMBIM6	SNTB2	KPNA2
KCNS1	FZD8	CFLAR	FAM125B	AKT1	SHC4	KAT2A		SEMA4G	PHKG2	FLNA	NOB1	PRND	MCM8	RASGRP3	C7orf43	ZNF426
NRGN	LEF1	RIPK1	PIP4K2B	AKT2	AKT2	ADAM17		ANGPTL2	TEP1	ZNF267	PDHA1	MRPL51	TPM1	C1orf21	ZSCAN29	HMBOX1
A_32_P85405	CREBBP	BAD	RUFY1	ARRB2	NRAS	RFNG		SEZ6L	NAA50	ATXN7L3B	ATXN2L	FBXO7	CBY3	DPP8	AZIN1	POLR3A
TRIM54	FZD9	IRAK4	SMAP2	CD14	AKT3	RBPJ		NUPL2	ENTHD1	CCNB2	FAM216B	NOTCH2NL	MMP16	PSAT1	DNMT1	MED28
GDA	PRICKLE1	BID	HLA-G	ARRB1	SH2B1	DTX1		VAV3	CD164	GNS	F9	MAPRE1	ZRANB1	TMBIM6	PLSCR1	YIPF4
TNNT2	CTBP2	BAX	FLT1	NRAS	NFKB1	CREBBP		SYAP1	DERL2	DSTN	DHX40	SERF2	COL4A1	GP5	RFX7	FHDC1
TESPA1	ROCK1	IKBKB	STAM	DUSP16	NFKBIB	DTX2		PNRC1	ZNF417	TNFAIP8	USH1G	BRD2	GABRB2	42986	TSPYL1	RAB42
KCNV1	CTBP1	CASP6	MDM2	CHP2	NFKBIA	MAML1		BCAN	PDPK1	CLTA	MOCS3	HSPA8	RACGAP1	RNF138	TAF4B	BLCAP
GALNTL5	WNT9B	IL1A	PIKFYVE	RASGRP3	NFKBIE	CTBP2		EFNB2	HSPE1-MOB4	INPP4A	CCDC12	RNF126	GIMAP1	42986	SOCS1	SERINC1
SLC30A3	WNT9A	AKT1	VPS37D	NFKB2	PIK3R5	NCOR2		SLC43A1	CD164	KIAA1279	CD274	PTDSS1	RB1	CCDC6	BECN1	TSR1
NPPA	CTNNBIP1	CASP3	PIP5K1A	NFKB1	MAPK14	CTBP1		PLCG1	MAP7D1	CNOT6	ERBB2	C17orf51	ZIC4	KCNJ10	PEG10	TM4SF5
SLC26A4-AS1	DAAM2	AKT2	VPS28	MYC	NGFRAP1	DVL3		TCF7L2	MOB4	LMNB1	ZNF746	AFF2	NRL	CRLF3	ADAM9	CNOT6
OR14I1	TBL1XR1	TNFRSF1A	CHMP2B	NFATC4	MAPK12	JAG2		CHORDC1	PTP4A1	ABCC4	KIAA0754	ADCY3	DUSP16	C8orf44-SGK3	PPTC7	DCTN6
FREM3	MMP7	AKT3	FAM125A	MAPK14	RELA	DVL2		FCRL4	ETS1	DIAPH1	ZNF154	RAB1B	KIF5B	ARPP19	RUNX2	HAS3
RXFP1	CER1	CHP2	PIP5K1B	FLNC	KRAS	NUMBL		HIST2H4B	ASGR2	ADD3	SF3B3	SMG1	POU5F1B	PHACTR2	NFAT5	HIP1
LOC646627	MAP3K7	ATM	RAB11FIP1	FLNA	SHC3	MAML2		IGFBP5	FAIM2	TLR6	ZNF772	SQSTM1	SLC35C2	42986	NIPBL	HIST1H2BJ
THEMIS	VANGL2	ENDOG	ACAP1	KRAS	TRAF6	KAT2B		KIAA1324L	GINM1	CEP57L1	SLC27A4	HOXA10	MSH6	SGK3	GTF2E2	TTC9
A_24_P153035	WNT2B	NFKB1	ARRB2	FLNB	NGF	DLL4		MAGEB4	EMC8	ATXN7L3B	PLA2G4F	EEF1A1	UGT2B4	AKT3	COPS7B	LSM3
A_23_P47546	WNT11	NFKBIA	AP2A2	PRKX	CALML5	PSENEN		DPF3	KNTC1	ABCC1	VPS13C	SREBF2	OBFC1	TMBIM6	LHFPL2	RECK
A_32_P105747	WNT10B	CAPN2	GRK7	TRAF6	BDNF	DLL3		RACGAP1	MSH6	TAB2	PAQR8	NDE1	ALDOA	KLHL15	LARP1	GTF2H2C
A_23_P124534	DKK2	PIK3R5	ARRB1	TGFB2	NGFR	DVL1		COL5A3	SLC39A1	VAV3	ULBP3	STOML1	ICK	ASXL3	GALNT1	ARL10
GALNTL5	LOC728622	IKBKG	AP2B1	DUSP1	SH2B3	CIR1		CACHD1	CXCL10	ZNF426	NUP98	ZNF174	RPN2	WDR26	RPS4X	RBBP6
TMEM155	CHP2	CAPN1	AP2A1	DUSP2	CSK	DLL1		HTR1F	POU2F1	GHITM	USH1G	RNF126	ZNF805	PSAT1	H6PD	SYNPO2L
TNNT2	AXIN1	IL3RA	EEA1	TGFB1	CAMK4	LFNG		RNF122	ZNF417	CUL4A	ZNF394	SRSF3	CCDC69	FGF2	PNN	GDF11
PP12613	AXIN2	IL3	GRK1	TRAF2	YWHAB	JAG1		TPPP	ETS1	PTPDC1	FBXL16	ELF4	AP4S1	42986	FAM104A	GOLGA2
KRT17	DKK4	RELA	CSF1R	BDNF	BCL2	MAML3		PLAT	TEP1	TNRC6A	INPPL1	ARL9	LSM14A	NCL	HABP4	FAM114A1
TMEM233	NFAT5	ENDOD1	SMURF1	TAB2	CAMK2A	HDAC1		HOXA10	CD164	LBR	ERLIN2	PCNA	GINS4	42986	PHF16	CSNK1A1
HSPB3	MYC	APAF1	LOC652799	ECSIT	CAMK2B	HDAC2		SERPINF2	CYP1A1	SORCS2	PIGA	TMX3	KNSTRN	C1orf21	FAM134C	MED16
ADTRP	SOX17	PRKX	SH3GLB1	TGFBR2	CAMK2D	NCSTN		UNC13D	POLD3	ZNF208	NAA30	USP30	NOX5	MPL	ARPP19	MKRN1
RXFP1	CSNK2A1	CSF2RB	VPS4A	DUSP7	FOXO3	DTX4		ABHD15	MAP7D1	CENPM	GYS1	PI4K2A	KIF5B	SCAMP1	ARID3A	BMP8B
ENC1	CSNK2A2	TNFRSF10A	ARFGAP3	TGFBR1	CAMK2G	MFNG		LSAMP	PPP2R1B	ALDOA	KRAS	PPIL4	SULT1B1	SMC5	KIAA0226L	MBNL3
ITPKA	NFATC4	TRAF2	DNM2	DUSP5	IRS2	RBPJL		DHODH	PPP2R1B	HNRNPL	ARL5B	ARHGDIA	MBNL1	N4BP1	ZNF200	GNB5
LHX2	CSNK1A1	TNFRSF10D	HSPA8	DUSP6	SORT1			MBNL1	FEM1A	SBF1	FAM167B	HAND1	TAB1	KLHL15	CD2AP	ZNF264
ABCC12	NFATC3	NGF	VPS36	DUSP3	CALML3			SRGAP1		G3BP1	XRCC6	MTMR3	HDGF	42800	PRMT7	PDE4C
NEUROD6	CSNK1E	TNFRSF10B	PARD6B	TGFB3	CRK			EPHA4		VAV3	THRAP3	TBCC	USP48	OTUB1	SERPINC1	UNK
SATB2	BTRC	TNFRSF10C	RAB31	PLA2G4E	SH2B2			ZDHHC20		ZNF117	UCK1	CRCP	NEURL1B	FGF2	KLF10	GLO1
HS3ST2	PRKX	MAP3K14	RAB11A	DUSP4	CRKL			ALPL		CMTM4	HNRNPA1L2	SAP18	YWHAZ	C18orf25	PGGT1B	PRC1
FEZF2	SKP1	IL1RAP	TRAF6	CACNG5	PLCG1			KANSL1		PLOD1	LSM10	DSCR3	LIMS1	TMBIM6	STAU1	GABPB1
A_32_P164393	FBXW11	IL1B	CHMP4A	CACNG4	PLCG2			PI4K2A		ZNF257	FAM216B	BTG2	LGSN	TEX261	PHF13	HAUS8
ENC1	RBX1	IRAK2	PDCD6IP	NF1	YWHAZ			TOR1AIP1		THUMPD3	SORCS2	TUBB	UGT2B4	C1orf21	PCGF5	MYPN
CRYM	CSNK2B	IL1R1	LDLR	PLA2G12A	ARHGDIA			SP1		MC2R	BMP7	TOB2	KLHL23	BCL2L11	SNAI1	DAZAP2
NPY	SIAH1	IRAK1	PIP5K1C	NFATC2	CALM3			CSDE1		SESN2	RPP14	THEM6	ERMP1	PSAT1	RAP1B	RTN2
ENC1	TBL1Y	TRADD	NEDD4L	RASGRP4	YWHAH			IRF2BP2		NHP2L1	PBX2	NDUFB11	RPS7	42986	PPP1R2	FEM1C
TAC3	WNT5B	PIK3R3	ACAP3	MAP3K2	ARHGDIB			HNRNPA2B1		CHUK	HNRNPUL2	UCHL1		TM4SF1	DNMT1	LIN52
NEUROD6	CCND1	BCL2	CHMP5	MAX	YWHAG			SPNS1		VCAM1	HNRNPA1	ACTA1		SUB1	STRIP1	ADAM9
SLN	CAMK2A	BCL2L1	ARAP2	DUSP10	YWHAE			C21orf62		STS	FAM193A	DGKD			FEM1B	WSB1
HTR2A	NLK	BIRC2	IQSEC2	FGF9	CDC42			ZDHHC20		EWSR1	TRPC4AP	CDKN1B			PBRM1	ZNF385A
KCNV1	CAMK2B	IRAK3	HLA-F	FGF8	SOS2			CXCL3		ZNFX1	PHF8	MICU1			MYO1E	CXorf38
FRMPD2	CAMK2D	PRKACA	MET	FGF7	PTPN11			C12orf5		PTGFRN	MAGEB4	GNB2L1			LCP1	APH1A
NPY	CAMK2G	PRKACB	HLA-E	FGF6	RAF1			FBXO31		DCP2	GRK6	TMEM167A			ARPP19	RNF115
PART1	PRKACA	PRKACG	ARAP3	FGF5	SHC1			CLNK		DHX33	NKRF	SPHAR			LRRC8D	ITGA2
EGR3	APC	PPP3CB	ASAP2	FGF3	RIPK2			PLAUR		LASP1	JMJD1C	KMT2D			EYA3	SLC35C2
HTR2A	PRKACB	TP53	RAB11B	FGF4	RHOA			SPDEF		HS2ST1	EPG5	ARHGEF6			PPP2R1B	CYB5R4
DLX1	PRKACG	PPP3CA	RAB11FIP5	FGF1	BAD			TECPR2		BSDC1	C1orf95	BTF3L4			WDR89	CYCS
HTR2A	WNT16	PIK3CA	ZFYVE20	FGF2	IRAK4			VASH1		TPCN1	KLK2	TAF11			ERLIN1	HABP4
HTR2A	DAAM1	PIK3CB	PDGFRA	PTPN5	PRKCD			PAQR7		PRPF6	PKM	TUBA1B			IREB2	FEM1C
TNNT2	CHD8	FAS	AGAP1	FGF21	MAPK9			CCDC43		BLOC1S2	MED18	AFF2			GNA13	IRAK4
ADCY2	FRAT1	DFFA	DNM3	IL1R2	MAPK10			ZBTB8B		VCAM1	FASN	ZNF283			RNF34	FEM1C
CREG2	CACYBP	CYCS	AGAP2	MAPK9	MAGED1			AGO2		WEE1	ABCF2	ANP32A			ALG9	CYB5A
KALRN	CCND2	DFFB	ARFGAP1	CACNA2D3	MAPK11			SOS2		ATG12	CIZ1	ACTG1			AZIN1	LATS2
RXFP1	NFATC2	PIK3CD	ARAP1	MAPK10	MAP2K2			ARHGAP6		HNRNPUL1	AKT1	FOXC1			BTBD7	ICA1L
MLIP	NFATC1	LOC651610	CHMP4B	MAPK11	MAPK13			ZDHHC20		RPRD2	RPTN	SLC35E2			ZMYND8	ADAT2
ZNF727	CCND3	PRKAR1A	CBLC	RASGRP2	MAP2K1			SOST		TBL2	GOSR2	CCNT2			EML4	ZNF12

KCNJ4	PLCB2	FASLG	ACAP2	PLA2G2A	BAX		TGFBR1		PPIC	SORCS2	NR2F2			POU4F1	C1orf50
CREG2	PLCB1	PRKAR2A	VPS25	MAP2K2	MAP2K7		PALM2-AKAP2		TNRC6A	PDE3A	CDR2L			SEMA6A	TMEM19
MLIP	CSNK1A1L	PRKAR1B	RAB22A	PLA2G4A	MAP2K5		CHCHD3		AARS2	PPP2R4	RAB4A			YOD1	FOXJ2
CAMK2A	PRKCB	EXOG	EHD1	MAP2K3	RPS6KA2		SH3PXD2A		PTK2B	MAT2A	MPI			NAP1L1	ACOX1
RGS4	PLCB3	PIK3CG	PLD1	MECOM	RAPGEF1		RPL3		DSTN	SRRM1	SEC24C			SOX12	CAPRIN2
ITPKA	PRKCA	AIFM1	PLD2	PLA2G5	RPS6KA3		XKR4		TMEM170A	PHF8	PCNA			NACC2	KCNA7
KRT14	PLCB4	NTRK1	ITCH	MAPK13	GRB2		HCAR2		EP300	PDE1B	RPS6KB1			SLC35C1	ARHGEF3
C1QL3	WIF1	PIK3R1	CDC42	MAP2K1	MAPK3		ATF6B		WDR54	WBSCR27	SHISA5			DDAH1	MCM7
HTR2A	PRICKLE2	PIK3R2	SH3GLB2	FGFR2	RPS6KA4		AP5B1		USP5	FAM26E	KRAS			RAB10	LCLAT1
LOC100129291	PORCN	CHP	PARD6A	MAP2K7	MAPK7		OPN5		RPRD2	GDE1	CMTM8			PPP1R12A	SUGP1
NGEF	RHOA	CACNA2D1	CHMP6	RASA2	KIDINS220		EDEM1		ECH1	MAZ	MEGF9			ERG	ZNF805
RGS4	FRAT2	CACNB1	VTA1	MAPK8IP2	RAC1		BZW1		CAP1	DCAF7	IGF2BP2			CEP350	LRRD1
A_32_P187143	PRKCG	COX8A	RNF41	RASGRF1	SOS1		TM4SF20		DOCK4	ZNRF2	THRA			WDR43	KLF3
HTR2A	MAPK9	CACNB2	DNAJC6	MAP2K5	MAPK1		SLC39A11		MPP6	SPTLC1	ZNF566			FOXA1	DIS3L
SATB2	MAPK10	UQCR10	ARF6	RASGRF2	RPS6KA1		RAB22A		MPV17	MSH3	SH3GL1			RGMB	BAZ1A
TBR1	WNT3A	COX7C	CHMP1B	FGFR4	YWHAQ		BCL2		GNB2L1	SSBP2	EIF5A			EPG5	MIXL1
RPRML	DVL3	CACNB3	PSD2	MAP2K6	HRAS		NOX5		CPEB3	PATZ1	EIF2AK2			CELSR3	PARP1
SLC17A7	RAC2	CACNB4	USP8	MAPK8IP3	MAPK8		ZNF48		BTLA	ACAP2	HYPK			MAST3	KCND3
TAC3	DVL2	CACNG1	PSD4	MAP3K6	PRDM4		BID		TPM3	CSDE1	MARCKS			APLN	SPRED1
A_32_P795813	RAC3	COX6CP3	WWP1	CASP3	MAPKAPK2		ZYG11B		RUNX2	VKORC1	AP3S1			ZDHHC20	PTGIS
SLC26A4	FZD3	FXYD2	HLA-C	MAP3K12	GSK3B		HARS		CRKL	POFUT1	GNL1			PHF13	COX10
ANXA8	DKK1	CACNG8	CXCR4	FGFR3	PSEN1		RAB40B		SEMA6D	UBE4A	SLC39A1			EPB41	HOXB3
MEF2C	CXXC4	RYR2	HLA-B	FGFR1	NTF4		SMOC1		FOXR2	CD9	MEF2D			DHX40	TTC8
A_32_P11425	DVL1	CACNG6	PRKCI	RASA1	IRAK2		NFATC1		ADAM28	EPHB3	HSPA12B			HIC2	UBXN2A
KLK7	FOSL1	ATP2A2	CXCR2	FGF14	NTF3		BCL2		C2orf69	RBM47	NUCKS1			TRIM59	SAR1B
MCHR2	CUL1	CACNG7	SH3GL3	RPS6KA2	IRAK1		BPTF		UBR3	KIAA0754	TCF25			AKIRIN1	SLC35F5
VIP	WNT10A	TPM2	CXCR1	RPS6KA3	RAP1A		TGFBR2		SRC	KPNA2	MTMR14			GNA13	ZNFX1
HTR2A	WNT4	CYTB	TSG101	FGF17	PIK3R3		PADI2		ZNF268	PSD3	FAM171A1			BTBD1	RABGAP1L
CAMK2A	SMAD3	TPM1	HLA-A	FGF16	RAP1B		SAMD1		TMED7	IHH	COLEC10			ZFAND5	SORCS2
MOXD1	TCF7	ATP1B2	ERBB4	FGF10	IRAK3		BRD4		PDCD6IP	TBL1XR1	GINS4			PGM3	PNRC1
HTR2A	SMAD4	ATP1B3	HSPA1L	GRB2	TP53		BEX2		UQCRC2	NOTCH1	SRRM2			PCNT	VPS26A
HTR2A	RAC1	ATP1A4	HSPA1B	FGF11	PIK3CA		VAV3		TOPBP1	SYNRG	PLK1			MED29	CREB1
LHX6	TCF7L2	ATP1B1	ERBB3	FGF12	PIK3CB		G3BP2		SERINC3	PEX5L	CASP16			GNPDA1	GALNT3
NEUROD2	SMAD2	COX2	HSPA2	FGF13	PIK3CD		INADL		RPL22	ULBP3	PLD3			TOMM5	FGF7
SLN	WNT1	COX7B	RAB11FIP4	PLA2G1B	GAB1		CHCHD5		RRAGD	ASXL2	ZBTB11			SLC4A7	ZNF578
RTP1	MAPK8	COX1	SH3GL1	RPS6KA1	FASLG		EID2B		ZNF273	KIAA1671	THEM6			ARID3A	TSR1
CRYM	EP300	COX7A1	EPS15	MAPKAPK3	SHC2		EZR		FAM91A1	TOMM5	ZNF280B			KPNA6	MFSD2A
FOXG1	WNT7A	COX7A2	DNM1L	IKBKG	ATF4		BMPR1A		NSD1	FAM127B	NELFE			SERPINE1	TFAP4
HTR2A	GSK3B	COX8C	HSPA1A	HRAS	IRS1		AGAP1		TCP1	GLI1	TMEM170B			XPO1	GATAD1
CABP1	WNT7B	COX6C	TFRC	CACNG2	ZNF274		SAMD5		ANKRD17	CNNM4	RNASEK			SERPINE1	MYH9
A_23_P200843	PSEN1	COX3	SH3GL2	FGF23	NTRK2		RAB40B		ZNF550	NOTCH2	TMEM30A			DLL4	C9orf40
SLC17A7	WNT8A	CACNA2D3	SMAP1	CACNG3	PIK3CG		ELP2		RPP14	C1R	LARP1			IER5	RBM41
ADCY2	WNT8B	COX6B1	RAB11FIP2	MKNK2	IRS4		SYNJ2BP		HMGA2	AR	ERF			STX17	EIF2S1
A_24_P153035	WNT2	CACNA1D	FGFR2	FGF18	NTRK1		ZNF398		AP4B1	BVES	CELF1			ELOVL5	C19orf40
SOWAHB	WNT3	SLC8A1	KDR	STK4	PIK3R1		PRDM2		QSER1	AGO1	PCNA			FBXO3	BLCAP
HTR2A	WNT5A	COX5B	KIT	STK3	PIK3R2		TBPL2		FNIP1	ARRDC1	KIAA1147			JOSD1	NR2F6
C2orf55	WNT6	SLC9A1	FGFR4	MAPK8IP1	CALML6		RBM48		NOL11	ZNF772	HMGB3			NDUFA12	FYCO1
C17orf96	CTNNB1	LOC644310	GIT2	MOS	NTRK3		SOX4		ZNF257	SMO	CYB561			RRM2	EFCAB11
SATB2	PPP2CB	CACNA1C	DNM1	RAP1A			SLC43A2		RPN2	CCND1	ATP5O			G3BP1	SNAP47
LOC339524	PPP2CA	TNNI3	FGFR3	MAPT			SHOX2		PTEN	SMO	TET1			VASH1	UBOX5
CABP1	PPP2R1A	CACNA1F	LDLRAP1	RAP1B			NTRK2		SSR1	GRWD1	ZFP91			RRAGD	REST
RSPO2	TBL1X	COX4I1	RET	PPP3CB			PRKAR1A		STARD7	SMO	ZKSCAN8			ENTPD1	DNAJB13
AQP9	PPP2R1B	ATP1A1	CCR5	PPP3CA			SNAI2		SLC16A1	HOOK3	STAU1			UBE3C	SLAIN2
HTR2A	ROCK2	CACNA2D2	PRKCZ	CACNA1H			ONECUT3		HERC3	HSPA1B	RNF8			TFDP1	HSPA4L
CHRM3	NKD1	CACNA1S	PSD3	CACNA1G			KIAA0754		RNF38	ACTB	PAPOLA			TMEM106B	USP12
MPPED1	FZD10	COX7B2	ASAP3	CACNA1I			TTC38		PLAG1		MTHFD1			PTP4A1	PTRF
VIP	FZD5	ATP1A3	HSPA6	ATF4			CDC42		HNRNPK		TPP1			NAA25	YOD1
HTR2A	NKD2	TNNC1	RAB5C	TAB1			THRB		TCF25		CSTB			CFDP1	GPRIN3
ICAM5	TCF7L1	ATP1A2	DAB2	FOS			FNBP1L		RPL7L1		CECR2			ZCRB1	FZD6
FHL2	RUVBL1	UQCRB	SMURF2	TAOK2			DUSP10		ZNF485		CDKN1B			KIF11	TNIP3
TYRP1	PPARD	COX6A2	HRAS	RPS6KA6			DNAJC30		ZNF669		CAMSAP1			ELOVL4	ZFYVE26
TBR1	PPP3CB	SLC9A6	CHMP3	PPP3R2			SEZ6L		DGCR2		PTP4A2			BAHD1	GATA6
OVOL2	TP53	TPM4	VPS37A	CACNG8			FARP1		ILVBL		AQP3			TAOK1	ZHX1
A_23_P200843	PPP3CA	ACTC1	EGFR	PPP5C			BLOC1S5		SPTY2D1		HIST1H3B			GIGYF1	TNRC6B
C3orf80	PPP2R5A	COX6A1	IL2RG	PPP3CC			GP2		SLC35G3		TUBB2B			SLC38A7	RNASEH1
TAC3	PPP2R5E	CACNA2D4	VPS37B	CACNG6			PDF		PTPN14		YOD1			AZIN1	ENTPD4
RGS4	PPP2R5D	UQCRH	IL2RB	PPP3R1			SERINC3		ZNF704		ACBD3			S100PBP	ZFYVE26
CYP26A1	PPP2R5C	UQCRFS1	CBLB	CACNG7			MRPS27		RAB5B		CCND2			SMAD1	PDHB
CACNG3	PPP2R5B	UQCRC2	CBL	MAP2K4			STRBP		HMGB2		RELA			MFAP3	IFFO1
KCNF1	FZD2	TPM3	PSD	ATF2			ZNF362		CELF1		FAM127A			PNMA1	HIST1H2BG
HTR2A	SFRP5	UQCRC1	NEDD4	PDGFRB			PRICKLE4		TOR1AIP1		CHD8			SIX1	PBK
MEF2C	SFRP4	CACNG2	EGF	JMJD7-PLA2G4B			ZFHX3		RPRD2		THRAP3			MTR	CNKSR3
CABP1	CHP	CACNG3	PARD6G	MAP4K3			ADORA3		C11orf68		SIGMAR1			IKZF4	FBXL7
HRH1		UQCR11	SH3KBP1	PLA2G6			SMAD4		SOS1		AGO3			VPS33A	MORC1
HTR2A		UQCRQ	LOC652614	PLA2G2E			SLC38A7		ZNF701		ZSCAN25			SRPR	NUGGC
CAMK2A		MYL3	ARFGAP2	PLA2G10			TMTC2		SERINC3		CASC3			KDM3A	FAM160B1
TEX29		TNNT2	RAB5B	MAP4K4			BCL2		DMTN		NFAT5			QRFPR	DEGS1
LHX6		MYL2	RAB4A	RPS6KA5			WNK3		TNRC6B		GINS4			IFNAR2	TXNIP
FAM19A1		LOC727947	EHD4	BRAF			SNAI1		MMGT1		AKAP1			RAB22A	RBBP6
FRMPD2		COX6B2	RAB5A	IKBKB			NPTX1		PRKACB		RPL35			SOCS3	RBBP6
HTR2A		COX4I2	AP2S1	PLA2G4B			NOTCH1		ITFG1		WASL			ZBTB38	PARP1
KIAA1239		COX7A2L	EHD2	MAP3K11			XRRA1		FAM168B		AGO1			EED	LGSN
HTR2A		COX5A	IL2RA	CACNA2D2			ATP6AP1L		NOTCH1		ECEL1			CBX3	CMPK1
PPEF1		CACNG5	EHD3	IL1A			HMX1		TRIM59		FAM213A			SP4	ESR2

SLC22A9		CACNG4	AP2M1	PLA2G2F				MEIS2		UQCRFS1		DNAJB9			MCFD2	MKNK2
CCKBR		UQCRHL	SNF8	DAXX				FAXC		CDC20		FAM122B			ADPRHL1	NR2C2
CACNG3		CYC1	SRC	AKT3				PRSS38		PI4KB		ORC2			CD2AP	CD83
CHRM3		MYH7	VPS4B	GADD45G				TMEM156		MED4		PRCC			B4GALT1	CRISPLD2
SLC26A4-AS1		MYH6	STAM2	FGF20				MTA3		HAPLN1		RPLP1			VPS41	KLHL21
SST		ATP1B4	CHMP2A	RELB				FZD1		LMAN2		C12orf4			LCOR	C14orf105
A_32_P24685			GIT1	MAPKAPK5				BACH2		TNRC6A		UBC			SOCS1	TTC9
PRKCB			GRK6	MAPK12				ZNF22		STK11IP		RC3H1			MKRN3	MYO1F
CHRM3			RABEP1	RELA				NUDT3		ZNF704		PNRC2			GFPT2	TIMM17A
HTR2A			VPS45	GNA12				SERP1		VAV3		MLXIP			PGPEP1	SATB2
A_32_P51005			RAB11FIP3	HSPA8				PADI2		RNF111		CHAMP1			SH3PXD2A	NIN
SERTM1			CLTA	HSPB1				SLC39A9		FUT4		RPS24			EML4	COX19
SATB2-AS1			CLTB	PTPRR				WWC3		CPSF3L		OTUD7B			RNF138	UBN2
A_24_P557355			CLTC	LAMTOR3				SMAD6		CATSPERB		HNRNPUL1			CCNE2	DRAXIN
NGEF			CHMP4C	GADD45A				RAB10		ZNF578		GATSL2			NAPG	COX10
STX1A			GRK4	NGF				IKZF2		SDHD		PHLPP1			PER2	HS3ST1
SERPINF1			PARD3	DDIT3				HLCS		CSDE1		USP33			BCL9	MKNK2
STX1A			GRK5	MAP3K14				SIRT1		CDK2		HADH			ZBTB39	KLHL21
LOC100290023			ASAP1	TAOK1				SERPINC1		RNMT		TMEM147			CSF1	RPIA
HTR2A			NTRK1	PDGFA				GPR45		ZEB1		LRRC57			SIX4	TAOK1
DUSP27			EPN1	FGF22				SNAI2		UNC45A		MRPL51			BLOC1S6	RASGEF1A
A_32_P164393				PDGFB				ST3GAL1		BCS1L		SLC25A3			JAK1	CCDC30
CHRM1				NLK				FOXC1		PRAP1		SEC16A			ST3GAL5	MYO1D
VIPR1				PDGFRA				NGDN		ZIK1		PGRMC1			NRBP1	MANEAL
A_24_P870921				PRKACA				PHKG1		POM121C		HUWE1			PARP1	CADM2
HTR2A				PRKACB				CEP97		PANK2		FAM72A			SLC7A5	CBX5
HTR3B				PAK1				VAV3		AMOTL1		NCBP2			PDCD10	FAM13B
SST				PRKACG				CDX2		STAT5B		UBL5			EDC3	BCL2L2
CHRM3				CRK				FAM83F		FBN2		FBXL8			CDC7	ETNK1
SOHLH1				CDC25B				SH3BP5L		SWAP70		TTC28			KIF5B	DDR2
CACNG3				CRKL				RPP40		PNRC2		TEX264			RPS27A	KIAA1841
ASB2				MAP3K13				TPPP		PSMD14		GORASP2			POLR3E	CSNK1A1
PTPN3				PLA2G2D				HAS2		SERINC3		TMEM184B			TNRC6C	C15orf40
A_32_P16323				CDC42				ANKFY1		PIGM		HIST1H2BL			AVL9	PFKP
FEZF2				RASGRP1				KLHL40		ZNF275		SYNPR			VAPA	ZNF12
A_32_P168561				CACNA2D1				KCTD11		MLLT6		MED18			TFDP1	ZNF12
C2orf55				CACNB1				SERPINE1		RPS27A		RNF41			CHD1	UBN2
KCNQ5				SRF				MDFI		ZNF208		MC4R			SETD5	THEM4
TMEM132D				CACNB2				SH2B3		PDIA6		PCNA			B3GNT5	RNF34
CABP1				SOS2				WISP1		HIF1AN		TXN2			TMED2	KIAA0513
CBLN2				CACNB3				SFT2D2		RPL19		DDX47			SH3GL1	ZNF7
HTR2A				CACNB4				MIER3		ADAM17		DNAJB4			SFXN1	SNX5
C3orf80				CHUK				ADAT2		CA2		AFF1			LDLR	GIGYF1
HTR2A				CACNG1				CD44		RMND5A		RPS27			BCL6	GBP3
CASQ1				PRKCB				NTRK2		PLAU		CENPF			TBPL1	ZNF317
SLIT1				RAF1				RORB		ZCCHC2		UBR4			PCDH10	ZBTB18
MUM1L1				PRKCA				ZBTB20		VAV3		GLUL			NAP1L1	ZYG11A
GPR26				TNF				AP1S2		USP31		YAP1			STX12	FZD6
C21orf128				PAK2				GIGYF1		ATF7IP		ATP5G3			POLRMT	CD28
MEF2C				MKNK1				ARNTL2		PNN		LRRFIP1			PPP3CB	MPP5
EPHB6				PLA2G3				MYRF		EN2		LETMD1			PLIN3	MBNL3
KCNS2				PRKCG				MYOCD		ARL6IP1		BCL2L2			IQCB1	TBC1D2
KRT16P2				PTPN7				BCL7A		ZNF257		SURF4			UHRF1BP1	SLC28A1
SERPINF1				RAPGEF2				BCL2		CLUAP1		NACA			RASGRP3	NHSL2
TEX29				HSPA1L				TGFBR2		MGAT5		HOXA10			TMED5	DNAL1
A_32_P36385				CACNA1A				FAM167B		NLGN4X		WBP11			PPP1R15B	ZBTB34
TRABD2A				HSPA1B				CPT1B		BRWD1		TMEM106B			ZNF770	WWC1
HTR2A				RAC2				HLA-DRB5		DDX27		CDKN1B			DYNLT3	DUSP2
NPTX2				HSPA2				POU2F2		MSH6		ZBTB38			MZT1	SUCO
A_24_P247774				CACNA1D				IL11		HS2ST1		RPL23A			ZNF264	PCGF5
SOHLH1				CACNA1E				AMOTL2		NUFIP2		SORBS3			SNRNP200	SOCS5
DLGAP2				RAC3				ZNF689		DONSON		SLC25A1			BAG4	PIGA
MCHR2				CACNA1B				HRK		ACTN4		VAMP8			SOX4	STAC2
COL5A2				HSPA1A				TCF12		ZNF485		HMGA1			ATM	ARID4B
CRH				PLA2G12B				DNAJB5		MARCKS		LEPR			USP37	LRIF1
PCSK1				CACNA1C				PRLR		C8orf33		GYLTL1B			FOXG1	ATAD2
LMO4				DUSP8				ETF1		ILF2		HOXA10			HIC2	APOH
NPTX1				CACNA1F				VAV3		MET		LNPEP			IFNE	SEPT2
PRKCB				CACNA1S				MAP1LC3<br>B		TNFAIP3		ETS2			MIER3	AGO3
RSPO2				MAP4K1				LRRC4		FAM222B		EIF1AX			MTRNR2L10	ELAVL2
NEK2				TNFRSF1A				ANKRD13<br>A		ZNF268		PHPT1			SRSF7	SNX5
LDB2				DUSP9				RNMTL1		NMT1		INTS1			RRM2	FOXRED2
ANO3				PPM1A				GLP1R		BCL2L1		CD4			ZNF507	BLCAP
A_32_P35106				PPM1B				SOWAHC		ARFIP1		PSMD11			SOCS1	SRCAP
LMO4				MAPK3				RPLP1		TNFAIP8		CAB39			SAE1	CMPK1
TRIM48				RPS6KA4				HOXC8		ZNF485		PROX1			PAWR	C9orf40
ICAM5				CACNA2D4				MYLK4		RPRD2					MRO	C9orf40
CRH				HSPA6				ABCC12		CD302					WDFY2	FZD9
GSG1				MAPK7				CLUAP1		PLAU					CHAT	ELK4
A_32_P232601				FGF19				ITPR1		MAP1B					PICALM	ZNF446
CDH9				RAC1				AKAP1		SERINC3					KBTBD6	FOXK2
A_32_P175935				SOS1				ENAH		ITPR3					IP6K3	C11orf54
C1orf115				MAPK1				SLC22A6		RBPMS2					SCML2	MYC
GLP2R				DUSP14				A1CF		DOCK4					PLXNA1	FOXK1

A_32_P79492				MAP4K2				ELMSAN1		NACC1					EIF2B1	RNF149
C14orf23				MAPK8				IL1B		DNMBP					PIK3C2B	KIAA1551
CHRM2				EGFR				ALDH6A1		ATP1A1					NACC2	PRRG4
C1orf115				MAPKAPK2				C17orf64		ZNF669					DCTN4	ZMAT3
PDE2A				EGF				GTDC1		RRAS2					PIK3C2B	PTPDC1
EPHB6				RRAS				TCF4		SLC6A15					PHTF2	DCTN5
NMU				TAOK3				AKAP13		GAPDH					ZNF589	KPNA2
DLX6-AS1				GNG12				MYO18A		E2F1					LARP1	LCLAT1
CHRM3				NTF4				VASP		RB1					EML4	TFAP4
NETO1				IL1B				PLAG1		DYNC2LI1					ANKRA2	PRIM1
KCNS2				MRAS				C11orf74		SSR1					UBXN4	RPRD2
RTN4RL1				NTF3				RAB22A		TSR1					PSMD7	LDHD
COL5A2				IL1R1				ADCY5		ZMYM2					OTUD4	SLC22A23
KLK7				TP53				TMPRSS3		AKAP11					PGPEP1	VPS53
RORB				FAS				RBM27		CRLS1					PIP4K2A	SNX5
ZNF831				NR4A1				CHRDL1		SSR1					UHRF1BP1	RBBP6
ZBBX				PLA2G2C				SLC1A1		ZNF704					SETD3	PDIK1L
A_32_P184636				FASLG				HSP90AA1		XPO1					TNRC6A	SBNO1
NNMT				NTRK2				PHF13		ETNK1					PCMTD2	PER1
CASQ1				NTRK1				RACGAP1		JMJD1C					CAT	HAS2
RPRM				CHP				MEX3A		SPTY2D1					ZNF460	LPAR2
KCNH5								WWC2		SYDE2					NDEL1	ISCA2
NMU								ARSE		HK1					MYBL2	TXNIP
A_23_P206120								MAP2K1		VCAM1					LRRC3C	GTF2H2C
PRKCB								AP1S1		KLF10					TPRG1L	LATS2
IQGAP3								SRCAP		KLHL2					CEP152	ZNF682
RTP1								HLA-DRB1		DDX47					LIFR	DPYSL2
SH2D1B								MKKS		ETS1					ZNF543	SLC35F6
ATRNL1								CEP97		MAP4					IL1A	CCDC125
FILIP1								IL1RAP		ABCF1					MYLIP	MINK1
KCNC2								AKAP2		DHX57					GNAI2	FBXL2
CCKBR								SLC27A2		ZNF485					ACER3	TIMM17A
CRLF1								GAN		ZNF578					NAP1L1	UBN2
LYZL4								FOXC1		EIF4A1					FRZB	KIF23
RASL10A								LY6G6D		GANAB					CREG1	PARP1
FBXO40								HCFC2		SNRPC					NAP1L1	CEP72
STX1A								UFD1L		NDUFV3					SRPR	ZNF347
A_32_P146597								AS3MT		ZNF138					SHC1	PRKACB
RHEBL1								SGPL1		SERINC3					MTRNR2L10	OCIAD1
GABRA5								TCF12		GNA12					AP2A1	MBD2
HRH1								ZBTB43		ZNF257					AFF4	DLGAP5
GYPE								CDH1		ERBB2IP					UBN1	TNFAIP1
A_32_P80455								RAB5B		MCFD2					RNF138	IFNAR2
LOC100287347								POU2F1		PRR14L					GLCE	KBTBD2
DOC2A								ATXN1		BCL7B					MTRNR2L10	TSPAN6
KLK7								CCNT2		ZNF701					KIF11	TSR1
PCDH8								SORCS2		CNN2					PLSCR1	F2RL1
KCNF1								MESDC1		RAF1					SBF1	FAM102B
PDE2A								IQCE		CEP104					PSMD7	HSPA13
CAMKV								DPF2		ZNF281					ASB3	TMEM14A
HS3ST2								RAB22A		NOTCH2					CAMKV	ZNF514
TRIM54								AP1S2		RAD51					FOXN2	NME6
CDH13								DNAJB5		CCT7					MLXIP	CPT1A
RHEBL1								ZNF705A		CCDC88C					MOGAT1	NR1D2
GABRA5								CDC42EP4		ZNF669					RNF220	USP15
SST								ETV3		ZNF208					FERMT2	LATS2
RFTN1								IL8		E2F4					FYCO1	MFAP2
DDN								AFF4		IMPDH2					ZFYVE20	UBXN1
FHL2								SH2D4A		PCMTD2					IKZF2	QSOX1
RASL11B								GALNT1		SOD2					MSANTD4	MCUR1
KCTD16								EZR		KIAA1210					GAB1	LIAS
DMRT3								LY6G6F		STARD7					WDR37	MKNK2
CHRM3								FZD8		JAK1					MARCKSL1	GTF2IRD2
LRRC8B								UBE2J1		ZNF704					METTL21D	NFIB
MEIS3								ALDH1A2		ACSS3					CPEB4	OSTM1
COL24A1								SOD2		ATXN7L3B					LMBR1L	CD44
A_24_P49647								CREB5		SUPT7L					KBTBD6	CRISPLD2
HTR3B								MRPS15		MED13L					TTLL12	DPP8
PKDCC								VIM		ZNF578					CSNK1G1	TNFAIP1
RBP4								RAB14		C8orf58					ATG12	IPP
RFTN1								ATXN7		KANSL1					N4BP2	PRPF4
A_32_P346000								MAFG		PPM1A					TXNDC5	GATAD1
LOC339524								IPO7		POM121C					LRRC8B	RGMB
GABRA5								IL1F10		HMGB2					ARID3A	RAB11FIP1
SLC17A7								MDFI		SOCS6					TP53	GBAS
RORB								C4orf3		SLC9A6					TFDP1	YOD1
PTGS2								FSTL4		ACAT1					STX16	BMP8B
A_32_P143778								USP47		ZNF281					SACS	C14orf119
MCHR1								ARHGAP2<br>9		HS2ST1					KIF5B	RAB22A
ST6GALNAC5								DENR		GTF2I					FBXO45	MYLK3
MET								ELP2		GJA1					IMPDH2	RASSF1
GPR26								ZSCAN22		MCFD2					MAPK8	EXO5
A_24_P504294								CDC23		SNTG1					MIB1	STX11
CRLF1								ITGB3		DNAJC21					SLFN5	HMGB1

OLFM1								ADCYAP1R1		SERINC3					JDP2	CRK
ISLR2								GTF3C3		PPARGC1B					HEY1	TMEM127
WIF1								DNAJC16		PRKACB					FRS2	COX10
MEF2BNB-MEF2B								TXNIP		VAV3					FAM8A1	ASB16
AKAP5								NUDT7		TNPO1					GOLGA1	ZNF532
NPTX1								AK2		BBS12					FANCF	DNAJB14
C19orf77								TTC39B		ATXN10					PPARGC1B	STK4
A_24_P703803								ZKSCAN4		ZNF253					CYB5B	KIF6
FAM81A								RACGAP1		MGAT5					MAPK8	DLGAP5
GAST								ELOVL6		COPA					ZDHHC17	CDKN2AIPNL
NETO1								LURAP1		PPP1CB					CLCC1	MARK2
FAM81A								BDNF		VAV3					GOLGA8B	UBE2V2
DLGAP2								RAB11FIP4		ATG12					ZNF543	MICA
PCDH20								CCR5		PSAP					ZNF646	RANGAP1
HTR1F								TPPP		DNAJC5					CAND1	CDK19
LZTS1								UTP14A		KDM1A					SYPL1	NR2F6
DLX2								HACL1		ZIC5					TGFA	MBNL1
AKAP5								BCAN		UQCRFS1					TWF1	MTMR9
TIAM2								MCTP1		ZNF138					SLC35G2	DUSP2
A_24_P910566								ZNF747		NPM1					AZIN1	GEMIN8
GRASP								FEM1B		HIST2H4B					PDGFRB	PFKP
CUX2								CACNA1C		ATXN7L3B					FOXG1	SPCS1
A_23_P139104								ATP2B1		S100A7A					PRKAR1A	CSDC2
SCARA5								CD28		MTAP					RPA2	HMGB1
ANKRD33B								MUC4		NACC1					IL21R	CUL3
FAP								C1orf64		CYB561D1					SEC24A	DKK1
HTR3B								HOXC8		VHL					KLF4	ACOT9
A_24_P781825								JARID2		ZNF485					AKIRIN1	ZNF785
EMX2OS								UGT2B28		ZNF275					MIA3	KPNA2
KHDRBS2								MRPS27		ATP5A1					SHROOM3	FZD9
RSPO2								CDH4		CDC73					PLEKHO2	SLC35F6
GABRA4								PLCXD3		ZNF682					RAB18	UBN2
CRYM								MED8		HMGCS1					DCUN1D1	MCM7
TOX								TLK2		PHF10					NCOA6	STX4
MUM1L1								TMEM236		CPSF7					GXYLT1	ZBTB33
A_32_P85360								MICA		VCAM1					B4GALT5	QRFPR
KCNC2								PLCXD3		ZNF550					PRDM1	TXNIP
DNAJC5G								IL11		WWP2					HMGN2	TNFRSF10B
RS1								SEC31B							LMBR1L	FITM2
KCNH3								TRPM3							CDCA7	DNAJC10
A_32_P84707								PTPRT							CDC37L1	COX6B1
GOLGA6L1								B4GALNT3							PTP4A1	SLC6A4
HGF								IKZF2							FAM8A1	KIF13A
KMO								CDH8							GNAZ	CCL5
LDB2								ENO1							GIGYF1	ZNF264
LOC375295								ZDHHC20							BDP1	SNAP47
GABRA5								LRRC55							CHST15	ZNF226
CLPSL2								SPOP							TRIM23	KCNMB1
LCE3C								SYT6							SOBP	ICMT
ATOH7								TSC22D2							SOX4	C4orf29
GABRA5								BCL2L2							BCL2	KIF13A
TMEM233								CCDC93							PSMD7	TRIOBP
ARC								WDR3							CREM	BACH1
PAH								ODF4							RNF122	WDR53
C2CD4C								RACGAP1							SOCS1	CMTM4
KCNH3								M6PR							ENPP4	NR2C2
LTK								ZNF623							LRP8	MAP1B
CPNE4								BBS9							PTP4A1	TXNIP
KCNH5								CACNG8							CSNK1G1	FKBP14
LOC646627								BCAN							MARCH6	WEE1
A_24_P76757								ASIC4							ADO	POLR3F
GRHL2								TMEFF2							IL21R	VEGFA
KIF17								TIAF1							OLFML2B	PRIM1
PTGS2								42795							LYPLAL1	CLCN3
SCN3B								SORCS2							TRERF1	TGFBR2
MUCL1								RUNX2							GCLC	CXorf38
DRD1								ASGR1							OPHN1	HOOK3
IGFBP2								SIX1							KLHDC10	CDIPT
GFRA2								PPP3R1							ACTR10	DAZAP2
PDZRN3								STC2							LARP1	BMP8B
C11orf87								ZBTB22							HIC2	PHC2
NOS2								USP14							CASP3	RAB11FIP1
NUDT4								TPPP							CSNK1G1	PDPK1
FAM19A1								KNTC1							PHF13	ZNF317
MEIS3								SNX9							ARF1	ANKS4B
STYK1								SPIB							B4GALT5	TIMM17A
CPNE5								LIPC							PGM3	TLR7
RASGRF2								BIRC2							SEC61A2	RABEP1
A_23_P413991								CAPRIN1							TNFRSF10B	RNASEH1
OCA2								C6orf132							ENPP4	GPSM2
LOC375295								VN1R1							KLF10	SERF2
LRRTM4								BCAN							ZNF507	CCDC6
OPN3								MAPRE2							CCDC71L	SLC35F5
HTR1A								CCRL2							REV3L	BLCAP

A_24_P840042							POP4								NOTCH1	MSH3
KCNG3							ARAP2								SP4	REST
FAM19A2							OPN5								TSHZ3	DAD1
KMO							ZNF385D								ATP5C1	HSPA14
A_32_P323							ZNF576								GIGYF1	MED16
A_24_P715434							SYNGR1								KLHL28	TNFAIP8L1
TMEM132D							DCAF7								LPCAT1	ATP1B3
NECAB1							IL1F10								CBX2	FAM73B
CHN1							SOX4								PAPD5	TRAF3IP2
GABRA4							ZCCHC24								RBBP7	FAM9C
SLCO2A1							PPP1CC								CCNF	SPIB
KCNC2							KIAA1244								ZNF567	TMEM9B
LMO3															GCSAM	MFN1
LCN15															CXCL11	LIMA1
SLC17A7															PHTF2	KPNA2
STEAP1															KLHL15	FAM213A
ANO3															KREMEN1	CMPK1
NR2E1															OTUD4	MELK
EXTL1															C8orf76	RAD23B
CDH13															PICALM	QSOX1
GABRA5															PDCD10	CECR2
CORT															RBM14	BTG1
GABRA4															STRN	NOL9
GABRA4															DDAH1	TFAP4
CCDC3															ROCK2	MAPKAPK5
GABRA5															TBC1D10B	ZNF264
NPFFR2															SP4	MCM7
SYT16															TAOK1	CD24
A_23_P104484															LCLAT1	NUPL1
KIAA1239															ATP2A2	HNRNPR
ATRNL1															PTAR1	IFNAR2
CHRM3															ATG12	SLC25A33
PHLDA2															FAM91A1	RGMB
DYDC2															BAZ1B	CEP104
COL24A1															HABP4	MTOR
PRSS16															USP53	MBNL1
FAP															LIN7C	CENPF
POU6F2															MTF2	FXYD5
HGF															ZNRF1	RPL17-C18orf32
SCN3B															SERBP1	SH3BP5
IL12RB2															FYTTD1	ARID4B
KCNT2															DCAF12	KCNK6
WDR16															FUCA1	SERF1A
KCNH7															FAM81B	F2RL3
HTR1F															DBF4	KIAA1919
GPR6															C1QBP	NEK8
C19orf77															HHIPL1	HADHB
A_23_P157027															GLCE	PCNXL2
GABRA4															C7orf60	FAM160B1
MAPK13															MSANTD4	TMOD3
PTPN3															HIC2	FGF7
NELL1															SKIL	TCEB1
A_23_P213527															MARCH4	PACRGL
MAL2															SH2B3	IPO9
OR14I1															NCOA3	KREMEN1
HTR1F															TANK	DAZAP2
HTR1F															KIF5B	DNAJB4
HTR1A															MIER3	KIAA0513
GABRA4															SP4	CHCHD5
BCL11A															APLP2	RAD52
A_23_P306240															PREPL	TNFAIP8L1
COL5A2															BECN1	LEFTY1
A_32_P233256															RAD23B	RFK
CRYBB1																TXNIP
CHRDL1																FAHD1
GABRA5																REST
GABRA4																EFCAB11
OLFM1																MYBL1
ERICH1-AS1																FZD9
HTR1F																FXYD5
ADAMTS3																PIGO
NELL1																GINS4
A_32_P156786																MSRB2
A_24_P384909																SIRT1
C13orf33																HABP4
MAFB																CFL2
RASL10A																FANCA
NOV																PSD3
GABRA4																SF3B3
LMO3																SUCO
CXCL14																C12orf49
CIDEA																SERF1B
GABRA4																TMEM100
SOSTDC1																SLC25A23

KLF10																GRK7
CBLN4																ABHD15
GFRA2																WDR73
ANKRD30B																ERGIC2
HTR1F																TIMM10
SHC3																ABLIM1
A_32_P168375																FEM1A
A_32_P156786																TEX30
SORCS1																HYPK
HTR1F																KCNJ8
GABRA4																FZD6
MAFB																DDI2
KIF17																FOXK2
A_32_P704328																ICA1L
TPBG																XPA
KCNH1																TXK
TC2N																CCSAP
A_24_P211106																NFIB
TIAM2																LY6G5B
NELL1																PFKP
MIR7-3HG																ZBED1
SLC26A4																CLIP4
GUCA1B																IFNAR2
DIO2																KLHL12
GABRA4																ELK4
HTR1F																NR2C2
ASGR2																MAN2B2
PP12613																KPNA2
BAIAP2L2																BCAS4
GABRA4																BTG1
PKDCC																CUL3
NECAB2																TUSC2
CPNE4																CRCP
NPTXR																C18orf32
A_24_P931017																RPL14
BCL11A																KIAA0513
THRB																C11orf1
FLJ42875																IGF1R
TC2N																SAMD8
KCNT2																C9orf78
HTR1F																PLA2G12A
DPP10																EXO5
GABRA4																RMND1
SYTL2																CSNK1A1
A_32_P138652																SLC7A11
ADRA1B																EMC1
VIT																DPF3
FAM212B																C12orf23
EMX2																GPR157
GABRA4																IRF2
RND1																KIF13A
GABRA4																CAPZA2
A_23_P206120																GINS4
KCNIP3																SEC23B
LRRC2																MRE11A
GABRA4																AGMAT
GYPB																BRI3BP
GRIN2A																FGF7
LOC644242																METTL8
GABRA4																TNKS2
GABRA4																KBTBD6
ZDHHC23																FZD6
DRD1																HMGB1
RTKN2																FXYD5
CAMKV																HERPUD1
MEPE																CEP72
CBLN4																GPR157
RASAL1																ABCG8
PRDM8																SIK1
LY86-AS1																GPR137B
HTR1F																LEFTY1
STYK1																ATP6V1C1
KCNH5																INSIG2
CCDC3																TMEM134
GABRA4																AGMAT
RARB																RNF19B
GABRA4																RAB22A
THEM5																BHMT2
KRT5																SUZ12
GRIN2B																SAMD12
GPR98																MICA
TAGLN3																KCNA7
LY6H																TNFRSF10B
KCNIP3																ABI2

PPEF1															CAPRIN2
FAM5B															SLC1A5
ATRNL1															HIP1
EPHX4															ESR1
AGBL4															ANKRD13B
PCDH20															GIGYF1
LNX1															INO80D
GABRA4															BLCAP
C4orf45															ZNF665
GABRA4															CREBRF
MKL2															CD44
BAIAP3															WDR53
WDR86															AVL9
GABRA2															LEFTY2
NCALD															APEX2
MYBPC1															MED17
CUST_3911_PI416261804															ZNF454
GABRA4															FEM1C
SYT16															POU2F1
FAM162B															PTRF
GCNT4															LUC7L2
MAST3															ZNF682
CIDEA															GIGYF1
PDYN															ZNF174
42983															DAZAP2
NPFFR2															ORAI2
NOS2															PFKP
MATK															FAM89A
SORCS1															GLTP
CUX2															MFAP2
KCNH7															RBM12B
SYT5															PAPD5
OSBPL3															ZBTB33
GABRA4															C12orf65
THRB															PAK2
MYBPHL															RHOC
RBP4															KDM1B
A_32_P219822															TMEM38A
NTN4															TXK
A_23_P40290															N4BP2L2
WDR16															BSCL2
AK5															SGTB
TWIST2															C5orf30
CXCL14															POLM
GABRA4															MARCH4
GABRA4															FOXK1
NETO1															HIVEP2
GABRA4															EIF2S1
GABRA4															GTF2H3
BCL11A															DAZAP2
A_23_P212781															ZNF554
LCN15															LATS2
GABRA4															POLM
KCNH7															ATP6V1C1
KIRREL2															PNRC1
RAB27B															PARD3
C2orf80															ULK1
FMN1															ADAM17
SPRN															
BCL11A															
KCNQ5															
CENPF															
SLCO4C1															
HOPX															
PPL															
NPAS1															
GABRA4															
EPHX4															
NPY1R															
GABRA4															
CHSY3															
ETS2															
TMEM132B															
PITPNM2															
FAM5B															
SEMA5B															
RASGRF2															
42983															
LRRC8B															
A_32_P106615															
FBXO40															
ADAMTS8															
RMI2															
ARHGAP32															

TRABD2A																
LAMB3																
GABRA2																
DRD1																
ANKRD24																
MYO5B																
MGAT5B																
ST6GALNAC5																
MCHR1																
A_24_P572303																
CDH13																
CCKBR																
GABRA4																
SSTR1																
DGKB																
FLRT2																
GABRA4																
GABRA4																
LRRC8B																
LRRTM4																
LRRTM4																
PLK2																
A_32_P75544																
CYP26A1																
GABRA4																
KCTD16																
TTC9B																
TNFAIP8L1																
RASAL1																
GABRA4																
DHRS7C																
CRHBP																
SLC22A10																
RBFOX3																
SYTL2																
HTR1F																
LOC285696																
OLFM1																
SLC17A7																
A_24_P374496																
R3HDM1																
GABRA4																
LOC643750																
PCSK1																
OLFML2B																
GABRA4																
PTGER3																
EFCAB1																
ARX																
SCN3B																
TOX																
EGR2																
AHRR																
GABRD																
GRIN2A																
PAK7																
GRIN2B																
USP43																
DUXAP10																
GABRA4																
POU6F2																
GABRA4																
MYBPHL																
KCNH1																
LRFN2																
TSHZ3																
PRSS16																
WIF1																
A_24_P66691																
C11orf41																
MAST4																
LINGO1																
A_23_P80759																
A_24_P927339																
A_23_P80759																
DUSP5P																
NPY1R																
SLCO1C1																
PCDH8																
FILIP1																
A_24_P788227																
OAF																
CAMK1G																
GABRA4																

CSGALNACT1																
FXYD7																
WNT7A																
ADAMTS8																
LRRK1																
LRRC7																
MIAT																
CDKL1																
MYO5B																
SMYD2																
MEPE																
CA11																
A_32_P46899																
ZBTB16																
HTR1F																
ARG1																
CA11																
A_24_P838797																
MICAL2																
LIPH																
GABRA4																
LY6D																
A_32_P68195																
VSNL1																
KLK5																
HTR1F																
B3GNT4																
LRFN2																
LMO7																
NPPA																
GABRA4																
COL5A3																
A_24_P627874																
FAM110C																
IER5L																
GABRA4																
A_24_P415195																
NPY1R																
SEMA3A																
PTPRO																
COL12A1																
NUDT4P1																
KCNN1																
SHISA9																
BAIAP3																
BHMT																
LOC158696																
CHSY3																
CDH22																
CACNG6																
IPCEF1																
RASGRF2																
ANKRD30B																
COL24A1																
LOC440084																
OLFM1																
TNN																
ARHGEF25																
A_32_P102581																
NPY1R																
CCNO																
GABRA4																
HRH2																
UNC5A																
GABRA4																
VSNL1																
CD163L1																
DIRAS2																
PLCB1																
FBLN7																
IPCEF1																
GABRA4																
C12orf42																
GABRA4																
A_24_P110625																
ANKRD30B																
CCDC68																
HTR4																
SYT16																
NPY1R																
C2orf80																
A_23_P76145																
RFX8																
DIRAS2																

BAIAP2																
DCLK1																
GABRA4																
SCN3B																
ZBBX																
ANKRD62																
UNC5D																
GABRA4																
NPY5R																
DUSP5P																
HS3ST4																
OR52K2																
PRKCB																
CD244																
PDP1																
PTGS2																
NPY1R																
EPB41L4B																
MORN3																
SHC3																
KIAA1045																
A_32_P41080																
VIPR2																
GABRA4																
SEC16B																
A_24_P911973																
PYDC2																
NR2E1																
DACH2																
TMEM132B																
GABRA4																
MKX																
DBH																
GPR98																
NCS1																
A_32_P171253																
NTNG2																
A_24_P153763																
SYNPO																
B3GALT2																
TYRP1																
PNOC																
A_32_P410272																
CSGALNACT1																
RGNEF																
ATP8B1																
EPHA3																
DIO2																
LOC154822																
A_32_P203232																
KALRN																
GABRA4																
RAB27B																
FAM84A																
TMEM132B																
KCNH4																
STX19																
A_32_P129265																
HTR1F																
A_24_P608007																
A_23_P80759																
TNNI3K																
PNCK																
UNC13A																
A_32_P42224																
A_32_P19966																
PCDH11Y																
HTR4																
GRIN2A																
A_24_P554040																
CHD5																
NPY1R																
PKNOX2																
GRASP																
SYTL2																
GABRA4																
SFRP2																
GRIN2A																
C8orf4																
GABRA4																
SLC35F1																
IGFBP2																
RAPGEF4																
CAMK1G																

ADRA1B																
GABRA4																
PRTN3																
A_23_P218295																
RTN4R																
FAM84A																
NTN4																
TNFAIP8L1																
CCND2																
ADRB1																
KIAA1644																
NUDT4P1																
STAT6																
HTR1F																
GABRA2																
LRTM2																
CAMKK2																
FAM49A																
NT5DC3																
LOC151009																
GLDC																
GCNT4																
NPY1R																
GABRA4																
AP1S1																
FXYD6																
HTR4																
LHX2																
A_24_P554040																
TAGLN3																
WBSCR17																
A_32_P74831																
NPY1R																
TSTD1																
A_24_P586072																
DUSP2																
RBM24																
DRD1																
LAMB3																
NELF																
SYTL2																
ASXL3																
A_23_P425210																
SPRN																
FMN1																
GABRA4																
SLC8A2																
NGB																
CYP26B1																
CDC6																
A_24_P940694																
NPY1R																
MIR7-3HG																
GJB5																
PTH2R																
MYBPC1																
MPPED1																
NUDT4P1																
DIO2																
EFNB3																
RBM24																
KCNB2																
PHACTR1																
A_24_P247576																
LOC100126784																
OTOGL																
MYB																
A_24_P840042																
NTSR1																
SPARCL1																
ART3																
GABRA2																
A_24_P347282																
TNFAIP8L3																
NTNG2																
A_32_P119348																
NUAK1																
ST6GAL2																
SOWAHA																
GABRA4																
A_32_P92188																
SLITRK5																
GABRA4																
GABRA4																

PRPS2																
GABRA4																
A_32_P123527																
EMX1																
SCAI																
GABRA4																
SEC24D																
SLC8A2																
BAIAP2L2																
PLK2																
A_24_P931211																
ARHGEF25																
KCNQ5																
HTR1F																
MICAL2																
CUX2																
RGS20																
DLGAP1																
KCNB1																
CPNE8																
GABRA1																
HTR3B																
ART3																
FBXW7																
DLGAP1																
NPY1R																
OXGR1																
C11orf87																
SLITRK1																
POSTN																
ST6GAL2																
CD52																
NPY2R																
RNF165																
RND1																
DGAT2																
LOC440895																
A_24_P840042																
KCTD1																
PTPRT																
FILIP1L																
PVRL3																
A_32_P17525																
DCLK1																
A_24_P924681																
GABRA2																
TSTD1																
OSTN																
HTR1F																
NTRK2																
PPP1R14C																
PLEKHA2																
GRIN2A																
SYT17																
NEURL1B																
A_24_P672240																
RHOBTB2																
CRHBP																
A_24_P787680																
KMO																
RFPL1-AS1																
HRK																
PLXNA1																
KCNG3																
ANKRD24																
KIAA1644																
A_32_P35047																
DACH2																
A_24_P903715																
GABRA2																
ARPP21																
PCDH11Y																
GABRA4																
NECAB2																
DUSP2																
LRRC7																
KITLG																
NPY1R																
RIMS3																
CHL1																
A_32_P101379																
ELFN2																
SOST																
KCNJ6																

GABRA4																
GPR22																
NELL2																
HOPX																
LOC100294469																
NPTXR																
A_24_P924351																
SMYD2																
NAGPA																
LOC151009																
BATF3																
SLC17A7																
RGR																
CTNNA2																
A_24_P564761																
CAP2																
HTR3B																
CNN1																
A_24_P937240																
PCDH11Y																
NECAB1																
C8orf46																
CHRD																
ST6GAL2																
VSTM2A																
DPF1																
KCNH7																
GABRA4																
GABRA4																
LNX1																
CACNB3																
GABRA4																
A_32_P114675																
DRD1																
GABRA4																
SLC39A10																
NUAK1																
TRIM51EP																
SERPINI1																
FAM162B																
PNOC																
RAPGEF4																
SNCA																
GABRA4																
F12																
C8orf46																
FLRT2																
TSTD1																
GPR98																
ADCY1																
LOC285501																
SMAD3																
GABRA4																
STK32C																
EPHA5																
A_32_P199220																
GRIK3																
LPPR3																
TMEM200A																
ARPP19																
RPH3A																
HTR3B																
TTC9B																
LOC643750																
B3GALT2																
GABRA1																
A_24_P861869																
EFCAB1																
GABRA1																
FOSB																
A_24_P924697																
CHST1																
DRD1																
MAPK11																
PHACTR1																
PTPRT																
PSD																
SERPINI1																
A_24_P838797																
GABRA3																
A_32_P207481																
OLFM4																
C21orf128																
CPNE8																

A_24_P582705															
SPON2															
ISLR															
GABRA2															
CNR1															
A_32_P171309															
NELL2															
LPPR5															
LOC285501															
ZNF184															
SYT17															
FAM131A															
TTC9B															
C1QL2															
PRSS35															
LRRC73															
MYOM2															
A_24_P563736															
GABRA2															
TNFRSF11A															
STAT4															
MMP17															
A_24_P926859															
ARNTL															
GABRA4															
BAI2															
TRIM51GP															
PRPS2															
TMEM244															
A_24_P738859															
DLX2															
PRDM8															
NPTX2															
CDH8															
LOC285696															
CCDC85A															
KCNMA1															
CCND2															
A_24_P509893															
SYT17															
NPY1R															
CRIP2															
MIAT															
SSX2IP															
NUDT4P1															
PRMT8															
TMEM200A															
FLJ32790															
KREMEN2															
RGS12															
TAGLN3															
EMID1															
KCNMA1															
CNR1															
CYP46A1															
A_32_P19262															
GABRA2															
A_23_P116195															
HTR2A															
WNT7A															
MFSD4															
ITPR1															
ANKS1B															
PAK6															
PRKCG															
A_24_P787680															
VSTM2A															
CDKL5															
RIMBP2															
HBQ1															
HOPX															
DYDC2															
MKX															
IQSEC2															
AGBL1															
EPHA4															
CDKL1															
GABRA4															
ZDHHC23															
ADAD2															
CADPS2															
CECR6															
GABRA1															
LARGE															

C1orf187																
HTR1F																
A_32_P140009																
ACTN2																
NUDT4P1																
TMEM241																
BCL11B																
EHBP1L1																
CADPS2																
KIF5A																
HOMER1																
PGM2L1																
CHGA																
KCNB1																
KHDRBS3																
MATK																
A_24_P376224																
HOMER1																
EPHA4																
MET																
EPB49																
KALRN																
SLIT1																
IL1RAPL2																
GRIN2B																
NUDT14																
PLCB1																
RNF165																
A_24_P365349																
SSTR2																
GABRA1																
GABRA4																
DCSTAMP																
TRIM48																
A_32_P113845																
MAL2																
CNR1																
CDC6																
A_24_P879722																
ADRA1D																
A_32_P16138																
A_24_P735306																
A_23_P137410																
UPP2																
ARHGAP32																
MCTP2																
GRIN1																
DRP2																
DPF1																
FBXL16																
FAM19A2																
SERPINI1																
PAH																
ATP6V1B1																
PSD																
MFSD4																
A_24_P910718																
PTPN3																
GABRA4																
MAEL																
SSTR2																
C4orf50																
A_24_P38316																
SYCE1																
LRRC73																
DRD1																
CORO1A																
NRG3																
GLRA3																
EGR4																
KCNG1																
GABRA4																
CRIP2																
CNR1																
A_24_P314571																
BTC																
CHML																
A_32_P145233																
GABRA4																
PRKCE																
GABRA2																
CDH8																
PCDH11X																
OTX1																

LRRC38																
SLC39A4																
PRSS3																
BAI2																
MGLL																
C1QL3																
EPHA5																
GABRA4																
C15orf27																
HAPLN1																
GABRA4																
NPY1R																
GABRA1																
PCDH7																
SYT1																
ABRACL																
BDNF																
NRN1																
FLJ42709																
A_32_P93115																
RGR																
GABRA2																
KCNMA1																
CHGA																
A_32_P230326																
LPPR3																
A_32_P198647																
LYZL4																
SEMA5B																
GABRA1																
GABRA2																
A_32_P133106																
GABRA1																
DUSP6																
CPNE4																
DPF1																
A_23_P352861																
CLSPN																
CHD5																
CAMK1D																
TDO2																
GABRA1																
RFPL1-AS1																
GPR22																
A_32_P47641																
TXLNB																
HTR1F																
SYDE2																
BEND5																
SCAI																
THRB																
OSBPL3																
RARB																
GABRA1																
C11orf41																
ZNF497																
DKK2																
NPY1R																
GABRA4																
ZNF697																
HTR1F																
FAM49A																
A_32_P145876																
LINGO2																
KITLG																
CCL28																
UBE2QL1																
MAPT																
SHROOM2																
DGKA																
GABRA2																
C19orf80																
GABRA1																
TSHZ2																
METTL21C																
OSTN																
AP1S1																
MORN3																
DENND3																
A_32_P199429																
WIPF2																
HS3ST4																
C11orf87																
GABRA4																

A_32_P203688																
PTPN5																
A_32_P214020																
KALRN																
NPAS2																
LPHN2																
PRKCG																
DPP10																
CNIH3																
A_32_P76255																
A_23_P86461																
CARTPT																
NPY1R																
GRIA3																
CACNB3																
A_24_P161173																
PAK1																
AGBL4																
SVOP																
SPINK2																
ZNF365																
SYNPO2																
LINC00473																
CDK1																
FAM211A																
AACS																
TAC1																
A_24_P545030																
RGS6																
A_24_P341626																
PCDH19																
A_23_P320407																
ASGR2																
TAC1																
SHANK1																
NPY1R																
TPM3																
PKD2L1																
HTR1F																
CNR1																
ZBTB16																
AACS																
GABRA4																
DUSP6																
LOC729683																
FAM131A																
SLC35F1																
PI4KA																
ABRACL																
FRAS1																
NGB																
EIF4E1B																
PTPRO																
CHRNA7																
CNTN3																
NPY1R																
CDH9																
A_24_P787680																
NPM3																
ADRA1D																
LOC643037																
CNR1																
CPNE9																
NICN1																
LINC00545																
PLA2G4A																
FZD8																
NPY1R																
VWC2L																
CD52																
LPPR5																
CPNE9																
CACNA1I																
MIAT																
TAC1																
WNT10B																
NLK																
KCNJ8																
CNR1																
TAC1																
GPR160																
GALNT9																
CRYGN																
SNTB2																

OVOL2																
GABRA1																
A_32_P35106																
NPY1R																
EHBP1L1																
DCLK1																
PDZRN4																
LOC400456																
PAK7																
C6orf222																
DUSP13																
PNCK																
EXD1																
EGR1																
PDZD4																
TAC1																
FAM131A																
ELAVL4																
OAF																
PCDH7																
A_24_P671561																
FLJ42709																
SLC6A20																
VSTM2L																
ADTRP																
KCNMB4																
AGBL4																
LOC643037																
EFHD2																
MMD																
FLJ42709																
GABRA4																
MGLL																
EPHA3																
A_32_P212483																
PLXND1																
ID2																
CACNA2D3																
DCHS2																
42798																
A_24_P707156																
SATB1																
PTER																
CDH12																
A_23_P384176																
PRR7																
GABRA1																
GLT8D2																
CHML																
MIAT																
CSMD1																
SH2D5																
LPA																
GABRA1																
KHDRBS3																
KCTD1																
TNFAIP8L1																
SLC22A10																
GABRA2																
MMD																
CCNYL1																
FILIP1																
COL23A1																
A_32_P18630																
ARNTL2																
GABRA2																
CLRN3																
GRPR																
KCNJ3																
GABRA1																
CNR1																
A_32_P168605																
HTR1F																
SEC24D																
GABRA2																
FLJ90757																
ERC2																
PRICKLE2																
AGBL4																
GRIK3																
ST6GAL2																
MYT1L																
PCDH7																
SYT5																

CNR1																
HABP2																
SEMA4F																
C9orf129																
RIMS3																
GABBR2																
CUST_8032_PI416261804																
LGALS2																
RASD1																
NPY1R																
CLSPN																
GABRA4																
A_24_P680704																
CNTN3																
PDYN																
RGS14																
A_32_P45229																
CNR1																
LOR																
POPDC3																
FANCA																
PTPRR																
DRD1																
SYCE1																
TRHDE																
PRICKLE1																
IGSF21																
EMX1																
KCNC3																
FOSL2																
SEMA3A																
ATP4A																
RTKN2																
STX19																
NEURL3																
GABRA1																
PKIG																
ACTN1																
ADCY1																
EGFL7																
A_24_P497235																
LOC100129321																
KIF21B																
FBXW7																
GABRA1																
KCNQ2																
ABHD12B																
STK32C																
GLRA3																
KIF5A																
CARTPT																
GABRA1																
RGS6																
GSTT2																
POC1A																
PDE6H																
CTXN1																
RPH3A																
CNR1																
DCBLD1																
TNNT1																
CACNA2D3																
A_32_P133106																
NLK																
A_32_P176036																
CNR1																
SYT5																
PCDH7																
FRAS1																
MYOM2																
GABRA4																
TAC1																
PHYHIP																
PTK2B																
FLJ42875																
SLC12A8																
PI4KA																
MSC																
MSX1																
ZMAT4																
TSPAN14																
CADPS2																
CX3CL1																
STAMBPL1																

TYRO3																
CCDC109B																
CDKL5																
LOC100131744																
DAPK1																
SYT13																
KIF21B																
KIAA1522																
HCN1																
ASXL3																
A_32_P99399																
GRIN3A																
CAMK1D																
DUSP6																
NOV																
PRDM2																
FAM153B																
IER5																
KDELR3																
FGF13																
PPP3CB																
SLC39A12																
CNR1																
GABRA4																
HLF																
CAMK1																
GAP43																
GABRA4																
C21orf62																
ANKRD30BL																
PCDH11X																
SBNO2																
CAMKK1																
A_32_P74763																
SLC44A5																
GNG2																
IL12RB2																
CNR1																
DUSP19																
STAMBPL1																
R3HDM1																
HGF																
CDKL5																
A_32_P78705																
PLXNA1																
A_32_P187349																
SLIT2																
LRRC4																
CARTPT																
GABRA4																
RSPH9																
CTXN1																
GAP43																
CNR1																
GSTT2																
P2RX6P																
A_32_P226200																
PHACTR1																
GRIK3																
LOC84856																
SOX5																
GNB4																
TAC1																
PTH2R																
HTR1F																
BASP1																
FAM65B																
NXPH2																
SCN2B																
ACTN1																
CNR1																
PTHLH																
ARHGAP32																
KIAA1751																
AP4S1																
CRYBB1																
TRIML2																
HSD11B1L																
HCN1																
UNC5D																
A_23_P76145																
CCNYL1																
PCDH11Y																
DCBLD1																

CDH10																
GABRA1																
GABRA1																
GABRA3																
NOV																
TAC1																
EFNB2																
GRIK3																
OSBPL3																
CAMK2N1																
PDE8B																
A_32_P192033																
A_32_P225021																
CTNNAL1																
CD3G																
TCERG1L																
LINC00473																
DCAF6																
DCLK1																
PLCH1																
RANBP3L																
CAMK1D																
GABBR2																
ATP8B1																
A_32_P5057																
KCNB1																
A_23_P215275																
GABRA1																
CCNYL1																
HTR1E																
GABRA4																
A_24_P459182																
WDR52																
SLITRK4																
PDE1A																
CNR1																
CNR1																
EFNB2																
CCDC85A																
FLJ42875																
FBXL16																
STAT6																
PARM1																
CNR1																
A_24_P871940																
LOC653602																
GABRA4																
CNR1																
GRM5																
ANKRD18DP																
PRKAR1B																
KCNJ3																
CNR1																
CENPF																
CYP46A1																
A_32_P103678																
CAMK2N1																
CALM1																
RPRML																
A_32_P1405																
GRM8																
GOLGA6L1																
SIRT5																
PCDHGC5																
EPHA10																
CPNE5																
BTC																
STAT4																
GPM6A																
TYRO3																
A_24_P290709																
CNR1																
ERC2																
TANC2																
CNR1																
FADS3																
RASD1																
A_32_P70245																
ST3GAL1																
A_24_P602168																
EPHA4																
GALNT9																
A_23_P212781																
GAS7																

BMPER																
BEAN1																
GABRA1																
NDFIP2																
DCAF6																
SYT1																
DKK2																
ASXL3																
DCBLD1																
CLSTN2																
XKR4																
CA7																
ANKRD19P																
PITPNM2																
BEND5																
STMN1																
FAM153A																
TPRXL																
LMO7																
ADAMTS3																
PRB2																
FRMPD4																
EFCAB1																
ERC2-IT1																
ELL2																
ARHGDIG																
DACH2																
BMP8B																
C5orf34																
ANXA11																
RGS20																
RAPGEF4																
CEP72																
PRKCB																
ACTN2																
FXYD6																
ENSAP3																
EPHA3																
ARF3																
ZNF365																
TAC1																
HOOK2																
FLJ42709																
DGAT2																
GAP43																
PITPNM3																
PLXNA2																
A_32_P83570																
PDZRN4																
CDCA5																
CDH12																
LOC284801																
SATB1																
SV2B																
DKFZP686I15217																
BAIAP2																
A_32_P11673																
FAS																
CAMK1D																
COL12A1																
STEAP1																
SLC4A10																
RASGRP1																
GABRA1																
RAB15																
BSN																
SYT7																
A_24_P76809																
ABHD8																
NEUROD1																
PTTG1																
FLJ30901																
CNR1																
HRASLS5																
GRIA3																
HGD																
SYP																
AHSG																
FUT1																
HAPLN1																
LOC613038																
MPND																
CUST_3621_PI416261804																
C12orf44																

TRIM36																
NAGPA																
GABRA1																
EHD3																
SLC44A5																
ARHGAP10																
GABRA1																
BIRC3																
IL36B																
WNT10B																
FAM65B																
GABRA1																
KIAA1217																
KCNA6																
EXD3																
FUT9																
A_32_P226556																
ARHGDIG																
GABRA2																
SV2B																
SYN2																
GABRA3																
RFPL3																
A_23_P371966																
FLJ42709																
CHIT1																
SLC39A4																
A_32_P142521																
DRD1																
TAF7L																
A_23_P12826																
MAP2K1																
FRMPD4																
GRIN3A																
MAN1A1																
HDAC9																
DCDC2																
LSP1																
ADCY2																
A_32_P47778																
WHSC1																
G6PD																
PSMG3																
LOC643037																
A_24_P393756																
CUST_4377_PI416261804																
CCL28																
KCNA4																
A_24_P85243																
A_32_P163891																
RPH3A																
ATP2B1																
RAP1GAP2																
CCDC3																
ARNT2																
CHST15																
PACSIN1																
ARPP21																
TRIM17																
SORCS3																
PPP3R1																
ERICH1-AS1																
NOL4																
GNA14																
CCDC68																
GABRA4																
A_32_P10083																
GPRIN1																
NDUFB3																
DLGAP1																
SNTB2																
ODZ2																
LINC00461																
SLIT2																
