# Supplementary material for: Downregulation of exosomal miR-204-5p and miR-632 as a biomarker for FTD: a GENFI study
Source: J Neurol Neurosurg Psychiatry. 2018 Feb 6;89(8):851–8. doi: 10.1136/jnnp-2017-317492 (PMC6045452; doi:10.1136/jnnp-2017-317492)

Supplementary figure 2

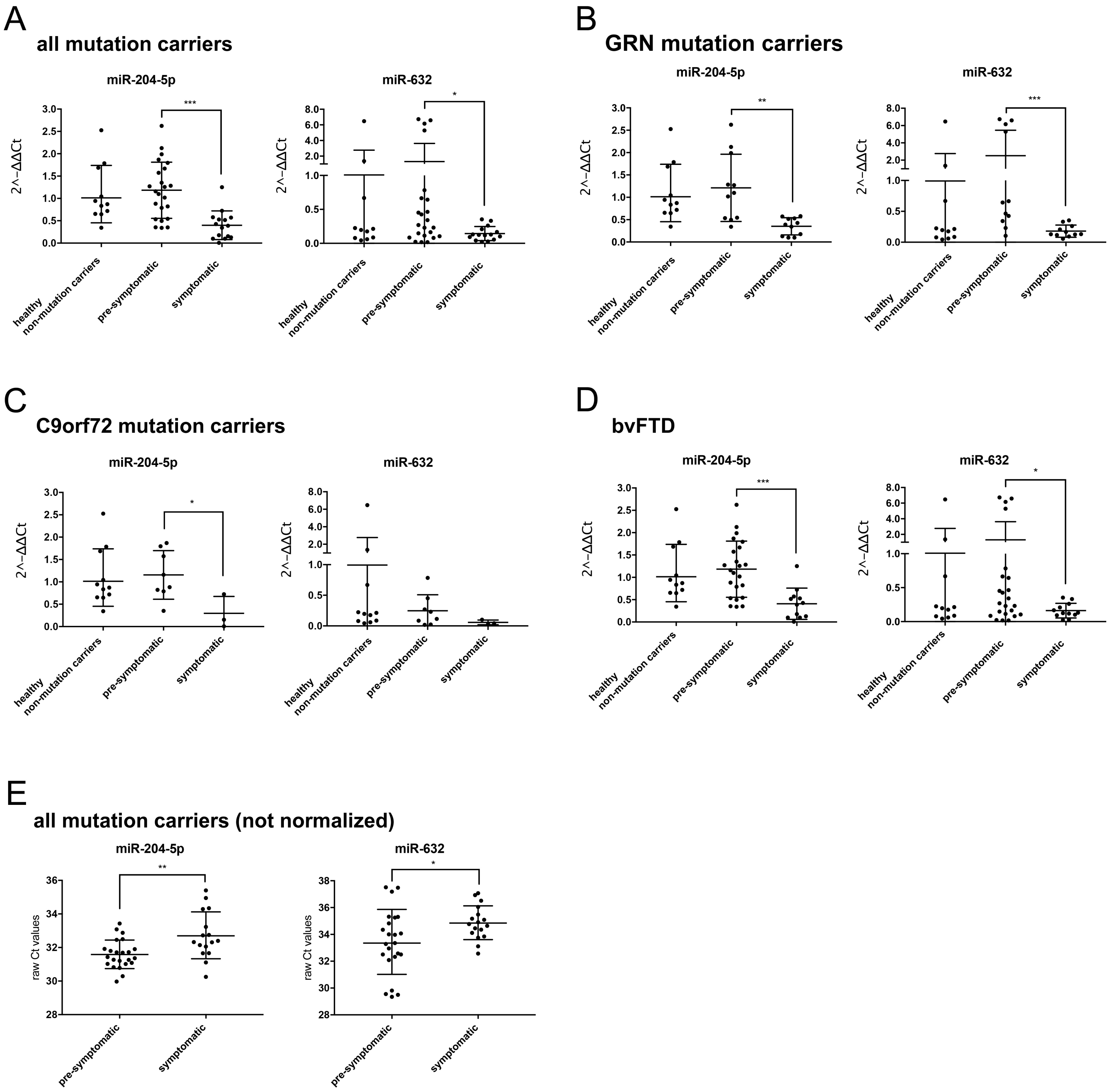

Supplement: Supplementary file 5 [file jnnp-2017-317492supp005.pdf]
